# Inflammatory Gene Regulatory Networks in Amnion Cells Following Cytokine Stimulation: Translational Systems Approach to Modeling Human Parturition

**DOI:** 10.1371/journal.pone.0020560

**Published:** 2011-06-02

**Authors:** Ruth Li, William E. Ackerman, Taryn L. Summerfield, Lianbo Yu, Parul Gulati, Jie Zhang, Kun Huang, Roberto Romero, Douglas A. Kniss

**Affiliations:** 1 Division of Maternal-Fetal Medicine and Laboratory of Perinatal Research, Department of Obstetrics and Gynecology, The Ohio State University, Columbus, Ohio, United States of America; 2 Department of Biomedical Engineering, The Ohio State University, Columbus, Ohio, United States of America; 3 Center for Biostatistics, The Ohio State University, Columbus, Ohio, United States of America; 4 Department of Biomedical Informatics, The Ohio State University, Columbus, Ohio, United States of America; 5 Perinatology Research Branch, Intramural Division, Eunice Kennedy Shriver National Institute of Child Health and Human Development, National Institutes of Health, Department of Health and Human Services, Bethesda, Maryland, United States of America; 6 Hutzel Women's Hospital, Detroit, Michigan, United States of America; Semmelweis University, Hungary

## Abstract

A majority of the studies examining the molecular regulation of human labor have been conducted using single gene approaches. While the technology to produce multi-dimensional datasets is readily available, the means for facile analysis of such data are limited. The objective of this study was to develop a systems approach to infer regulatory mechanisms governing global gene expression in cytokine-challenged cells *in vitro*, and to apply these methods to predict gene regulatory networks (GRNs) in intrauterine tissues during term parturition. To this end, microarray analysis was applied to human amnion mesenchymal cells (AMCs) stimulated with interleukin-1β, and differentially expressed transcripts were subjected to hierarchical clustering, temporal expression profiling, and motif enrichment analysis, from which a GRN was constructed. These methods were then applied to fetal membrane specimens collected in the absence or presence of spontaneous term labor. Analysis of cytokine-responsive genes in AMCs revealed a sterile immune response signature, with promoters enriched in response elements for several inflammation-associated transcription factors. In comparison to the fetal membrane dataset, there were 34 genes commonly upregulated, many of which were part of an acute inflammation gene expression signature. Binding motifs for nuclear factor-κB were prominent in the gene interaction and regulatory networks for both datasets; however, we found little evidence to support the utilization of pathogen-associated molecular pattern (PAMP) signaling. The tissue specimens were also enriched for transcripts governed by hypoxia-inducible factor. The approach presented here provides an uncomplicated means to infer global relationships among gene clusters involved in cellular responses to labor-associated signals.

## Introduction

The last three decades have seen prodigious growth in our knowledge of the basic principles of parturition in women. Indeed, we now understand that human labor in both the term and preterm settings is fundamentally an inflammatory process typified by the sudden, robust expression of pro-inflammatory cytokines (IL-1, IL-6, TNF-α) and chemokines (IL-8/CXCL-8, MCP-1/CCL-2) that elicit a panoply of downstream biological consequences culminating in expulsion of a viable offspring [1]–[3]. Matrix metalloproteinases (MMPs) are released locally by infiltrating leukocytes (neutrophils and monocytes/macrophages) that modify the extracellular milieu within the cervix and at the maternal-fetal interface [4]. Furthermore, cytokines secreted into the intrauterine microenvironment through the induction of prostaglandin synthase-2 (PTGS2) provoke the precipitous production of prostaglandins (PGE_2_ and PGF_2α_) that then stimulate myometrial contractility and cervical changes that must take place in order to deliver the fetus [2], [5]–[7]. A collection of coordinately regulated genes (contraction-associated proteins, CAPS), including the oxytocin receptor (OXTR), connexin-43 (CX-43), the prostaglandin F receptor (FP), and PTGS2 (also known as cyclooxygenase-2, COX-2) are dramatically increased during the early stages of labor [8].

Some of the molecular mechanisms that regulate these genes have been investigated, and we now understand that the inflammatory response during parturition is controlled, at least in part, by the master transcription factor nuclear factor-kappaB (NF-κB) [9], [10]. For example, we and others have shown that cytokines elicit the up-regulation of (PTGS2) for the synthesis of PGE_2_ and PGF_2α_ through the activation of heterodimeric (p65/p50) NF-κB and its inhibitor IκB-α in a variety of *in vitro* culture preparations of intrauterine cell types [11]–[15]. Furthermore, the genes encoding many cytokines (e.g., IL-1β and TNF-α), chemokines (e.g., IL-8) and MMPs (e.g., MMP-2, MMP-9) are themselves driven from NF-κB-binding promoters [16], [17]. Most of this information has been obtained by studying one or a few biomolecules in any given body of work.

Experimental reductionism has yielded many important and interesting insights into how a given gene (or gene product) might participate in mammalian parturition. Indeed, today we know far more about the underlying cellular and molecular participants that mediate uterine contraction and the biochemical events of cervical dilatation and effacement in humans than we did just a few years ago. The endocrine signaling of parturition that takes place via the hypothalamic-pituitary-adrenal-placental axis in the sheep [18], and the role of the corpus luteum in sustaining rodent pregnancy and the consequences of its regression on birth [8] are now understood in significant detail. Yet, collectively, this information has not substantively improved our ability to prevent, diagnose or arrest preterm labor in women to any significant degree. Nor do we yet fully appreciate the molecular nuances that distinguish term and preterm labor and birth.

A reductionist approach permits the intricacies of a given biochemical process to be unraveled, but the inherent quest for simplicity precludes an assessment of potential complex interactions that may exist between several molecular pathways. Increasingly, networks of interacting pathways are evaluated by examining a myriad of individual genes expressed under a given set of experimental or clinical contexts. In this regard, Romero and colleagues recently reported that mRNAs isolated from cells of the fetal membranes in women in spontaneous parturition at term manifest a characteristic “inflammatory gene signature” when compared to a cohort of women prior to the onset of active clinical signs of labor [19]. A similar pattern of gene expression was unveiled by this group in the uterine cervix in association with labor [20]. These studies offer the intriguing opportunity to investigate the complex molecular interactions that occur as the uterus and cervix transition from quiescence to active labor.

However, we know clearly that the onset of labor is not a binary switch that is suddenly unleashed; rather, it is a series of subtle biochemical and physiological epochs that arise in the last several weeks of gestation [21]–[23]. Therefore, the inability to assess this protracted time-course in women due to ethical realities renders this transcriptomic approach in women scientifically very challenging. Thus, in the present work, we have assembled a reductionist strategy using monolayer cultures of human amnion mesenchymal cells as a means to model the inflammatory gene expression signature previously reported by Haddad *et al*. [19]. Using a systems approach, we have examined the complex interactions that occur between networks of genes expressed preferentially following cytokine challenge in amnion cells. In addition, we have used computational methods to investigate the control of many of these genes by transcription factor binding motif analysis. The results from this simple cell culture preparation when compared to the data reported by Romero and colleagues offer a novel translational strategy for a more thorough molecular appreciation of human parturition in the term and preterm settings.

## Methods

### Cell Culture

Primary cultures of human amnion mesenchymal cells were prepared from amnion membranes obtained prior to labor at term, as previously described [12]. Cells were cultured in DMEM (25 mM glucose) supplemented with 10% fetal bovine serum, 2 mM L-glutamine, 1 mM sodium pyruvate, 50 μg/ml of gentamicin sulfate, and 0.5 μg/ml of amphotericin B at 37°C in a 5% CO_2_ atmosphere. Cell culture media, supplements and sera were purchased from Invitrogen (Carlsbad, CA).

### Treatment and RNA Extraction

Recombinant human interleukin-1β (IL-1β) was obtained from R&D Systems (Minneapolis, MN). Early passage cells at confluence were treated with IL-1β (10 ng/ml) for 1 or 8 h, whereas control cells were treated with medium only. Total RNA from control and experimental groups were extracted using Trizol reagent (Invitrogen, Carlsbad, CA) and the RNeasy Mini Kit (Qiagen, Valencia, CA) as previously described [12], [24]. Experiments were repeated three times for a total of nine samples used for microarray analysis. Three biological replicates were used to ensure that any results were not biased by day-to-day variation in RNA extraction.

### Microarray Analysis and Data Processing

Quantification and quality of total RNA samples were evaluated with the 2100 Bioanalyzer (Agilent Technologies, Foster City, CA). Three sets of RNA samples including vehicle control, 1 h and 8 h post IL-1β treatment were hybridized to GeneChip Human Genome U133A 2.0 Array (Affymetrix, Inc., Santa Clara, CA). All arrays were processed using the GeneChip System (Affymetrix, Inc., Santa Clara, CA) at the OSU Comprehensive Cancer Center Microarray Shared Resource facility following the manufacturer's protocol. The R statistics package version 2.7.1 [25] was employed for microarray data analysis. First, background correction and quartile normalization were performed to remove technical bias, and gene expression levels were summarized over probe set using the Robust Multichip Average (RMA) method [26]. These data have been deposited in NCBI's Gene Expression Omnibus [27] and are accessible through GEO Series accession number GSE26315 (http://www.ncbi.nlm.nih.gov/geo/query/acc.cgi?acc=GSE26315). A filtering method based on percentage (7 out of 9 chips) of arrays below noise cutoff (log_2_ scale of 6 expression level) was applied to filter out probes whose expressions are not detectable. Analysis of variance (ANOVA) modeling was performed and t-test was used to detect differentially expressed genes. To improve estimates of variability and statistical tests for differential expression, variance shrinkage methods were employed for this study [28]. The significance level was adjusted by controlling the expected number of false positives for all comparisons [29]. After these statistical analyses, significant genes were filtered from the three sets of RNA samples using the criteria of a ±2.0 fold change and a p-value of ≤0.05 for comparisons among the vehicle, 1 h and 8 h time points. A final list of genes was generated by eliminating duplicate probe sets and averaging the expression values of genes with multiple probe sets. The final lists of genes were subjected to subsequent clustering, time series, and transcription factor binding motif enrichment analyses as described in their respective sections below. For functional pathway and network analysis, the fold change (±2.0) and p-value (≤0.05) cut off, and probeset averaging for genes were completed directly with the Ingenuity Pathway Software. A heatmap representing the gene lists was generated with hierarchical clustering (Euclidean distance and complete linkage) using the MultiExperiment Viewer (MeV) software, part of the TM4 microarray software suite [30]. Short Time-series Expression Miner (STEM) [31] was used to identify temporal expression profiles, ranked by the number of genes fitted to each profile.

### Transcription Factor Binding Motif Enrichment Analysis

NCBI reference sequence mRNA accession numbers for each unique, significantly expressed probeset from our microarray analysis, as well as the published full-thickness fetal membrane data [19], were curated by conversion from Entrez Gene IDs and/or Human Genome Organisation (HUGO) gene symbols using g:Profiler (http://biit.cs.ut.ee/gprofiler/) [32]. Multiple probesets mapping to identical genes were consolidated based on unique gene symbol. Genes with multiple reference mRNA sequences were resolved by using the accession number for the longest or most frequently appearing transcript. The RefSeq mRNA accession numbers were then subjected to transcription factor binding motif analysis using the web-based software Pscan (http://159.149.109.9/pscan/) [33]. The JASPAR [34] database of transcription binding factor sequences was utilized for the analysis on position −950 to +50 of the 5′ upstream promoters. The range of −950 to +50 was selected out of the available range options in Pscan to best cover the range of interest of −1000 to +50 bp. Hypothetical proteins, pseudogenes, and expressed sequence tags lacking reference sequences were not included in the transcription factor binding motif analysis. Similar conditions and exclusions were used to perform promoter scanning analysis with the dataset of Haddad *et al.* [19]. MATLAB 2009 (MathWorks, Inc., Natick, MA) was used to generate a colormap showing the frequency of transcription factor motif occurrences, while Graphviz Editor version 2.26.3 [35] was used to generate a gene regulatory network map based on results of the transcription factor binding motif analysis.

### Functional and Pathway Analyses

Network pathway analyses were generated using Ingenuity Pathways Analysis (version 8.5, Ingenuity Systems, www.ingenuity.com). A full list of the normalized, filtered data set from the 9 microarray chips were uploaded into Ingenuity for analysis. For the Haddad *et al.* dataset, the microarray data taken from the published data were uploaded into Ingenuity for analysis. The genes from each dataset were set as molecules of interest which interact with other molecules in the Ingenuity Knowledge Base (identified as “Network Eligible molecules”). For network generation, the molecules from the normalized, filtered microarray dataset were each mapped to their corresponding object in Ingenuity's Knowledge Base. A fold change cutoff of ≥2 (log_2_-fold change cutoff of 1.0) was set to identify molecules whose expression was significantly differentially regulated. Multiple probesets that map to the same gene were averaged (preliminary analyses of individual probesets compared to multiple probesets representing the same gene demonstrated the validity of this approach). Network eligible molecules were mapped onto a global molecular network developed from information contained in Ingenuity's Knowledge Base. Networks of Network Eligible Molecules were then algorithmically generated based on logical connectivity. In the graphical representation of networks, molecules are represented as nodes (filled shapes), and the biological relationship between two nodes is represented as an edge (line). All edges are supported by at least 1 reference from the literature, from a textbook, or from canonical information stored in the Ingenuity Pathways Knowledge Base. Human, mouse, and rat orthologs of a gene are stored as separate objects in the Ingenuity Pathways Knowledge Base, but are represented as a single node in the network. The intensity of the node color indicates the degree of up- or down-regulation (red and green, respectively). Nodes are displayed using various shapes that represent the functional class of the gene product. Canonical pathways analysis identified the pathways from the Ingenuity Pathways Analysis library of canonical pathways that were most significant to the data set. The significance of the association between the data set and the canonical pathway was measured in 2 ways: (1) A ratio of the number of molecules from the data set that map to the pathway divided by the total number of molecules that map to the canonical pathway is displayed; and (2) the Benjamini-Hochberg method used to focus on the most significant biological functions associated with the dataset by adjusting calculated p-values that determine the probability that the association between the genes in the dataset and the canonical pathway is explained by chance alone.

Biological functional analysis identified the biological functions and/or diseases that were most significant to the dataset. Molecules from the dataset that met the fold change cutoff of 2 and were associated with biological functions and/or diseases in Ingenuity's Knowledge Base were considered for the analysis. Benjamini Hochberg method was used to calculate a p-value determining the probability that each biological function and/or disease assigned to that data set is due to chance alone.

### Quantitative Real Time PCR Analysis

Reverse transcription was carried out with 2 μg of total RNA using oligo primers and Superscript III reverse transcriptase (Invitrogen, Carlsbad, CA). TaqMan Universal PCR Master Mix and TaqMan Gene Expression Assays (Applied Biosystems, Foster City, CA) were used for quantitative real time PCR (qRT-PCR) with β-actin (ACTB) as a control. The following Applied Biosystems (ABI) primer/probe sets were used: *Nfkbia*, Hs00153283_m1; *Rela*, Hs00153294_m1; *Il1b*, Hs01555413_m1; *Il6*, Hs00985641_m1; *Il8*, Hs00174103_m1; *Cxcl2*, Hs00601975_m1; *Ptgs2*, Hs00153133_m1; and *Actb*, 4333762F. Quantitative RT-PCR was performed on a 7300 Real-Time PCR System (Applied Biosystems, Foster City, CA) using recommended cycling conditions and associated SDS software, and subsequent analysis with the comparative C_T_ method [36] was conducted using Microsoft Excel. Plots were constructed and statistical analysis was completed using GraphPad Prism software (La Jolla, CA). Statistical analyses were performed using the Kruskal-Wallis statistical test with *post-hoc* testing using Dunn's multiple comparison test when appropriate, after it was determined that gene amplification data exhibited a non-Gaussian distribution.

## Results

### Temporal transcription profiling of cytokine-stimulated amnion mesenchymal cells *in vitro*

Interleukins-1α and -1β (IL-1α and IL-1β) are encoded by separate genes, but share nearly identical biological activities. The pro-inflammatory response to either is elicited upon activation of a common interleukin-1 receptor (IL-1R), a heterodimeric complex comprised of the IL1R1 and Interleukin-1 receptor accessory protein (IL1RAP) gene products [37], [38]. The constituent IL-1 receptor proteins are themselves members of the IL-1R/Toll-like receptor (TLR) superfamily [37], [38]. As a result of conserved intracellular domains among these receptors, the signaling cascades elicited by IL-1α or IL-1β are generally similar to those activated by TLR ligation by viral and bacterial pathogens, with some exceptions [38], [39]. Therefore, the genes upregulated in response to IL-1β have broad implications in the context of innate host defense and, importantly, can mimic the initial response to bacterial pathogens in a microbe-free environment [40].

In the current experiments amnion mesenchymal cells (AMCs) were chosen as a relevant *in vitro* model system in which to study the inflammatory component of labor. We have previously reported that basal release of PGE_2_ in AMCs is 5-fold higher than induced release of PGE_2_ from amnion epithelial cells [12]. Our laboratory previously used this model to examine the induction of selected gene targets, such as prostaglandin E synthase (*Ptges*, also known as microsomal prostaglandin E synthase-1, mPGES-1) and prostaglandin-endoperoxide synthase 2 (*Ptgs2*), in response to IL-1β [12]. In addition, AMCs are a non-transformed cell model, which eliminates from consideration the strong affect of oncogenes and other factors that might influence the global transcriptional response [41], [42]. It was also reported that the epithelial-to-mesenchymal cell ratio was 4.3 to 1 at preterm (15 cases at 23 – 36 weeks) and 7.8 to 1 at term (27 cases) [43], suggesting a higher population of mesenchymal cells in the setting of preterm labor.

**Figure 1:**
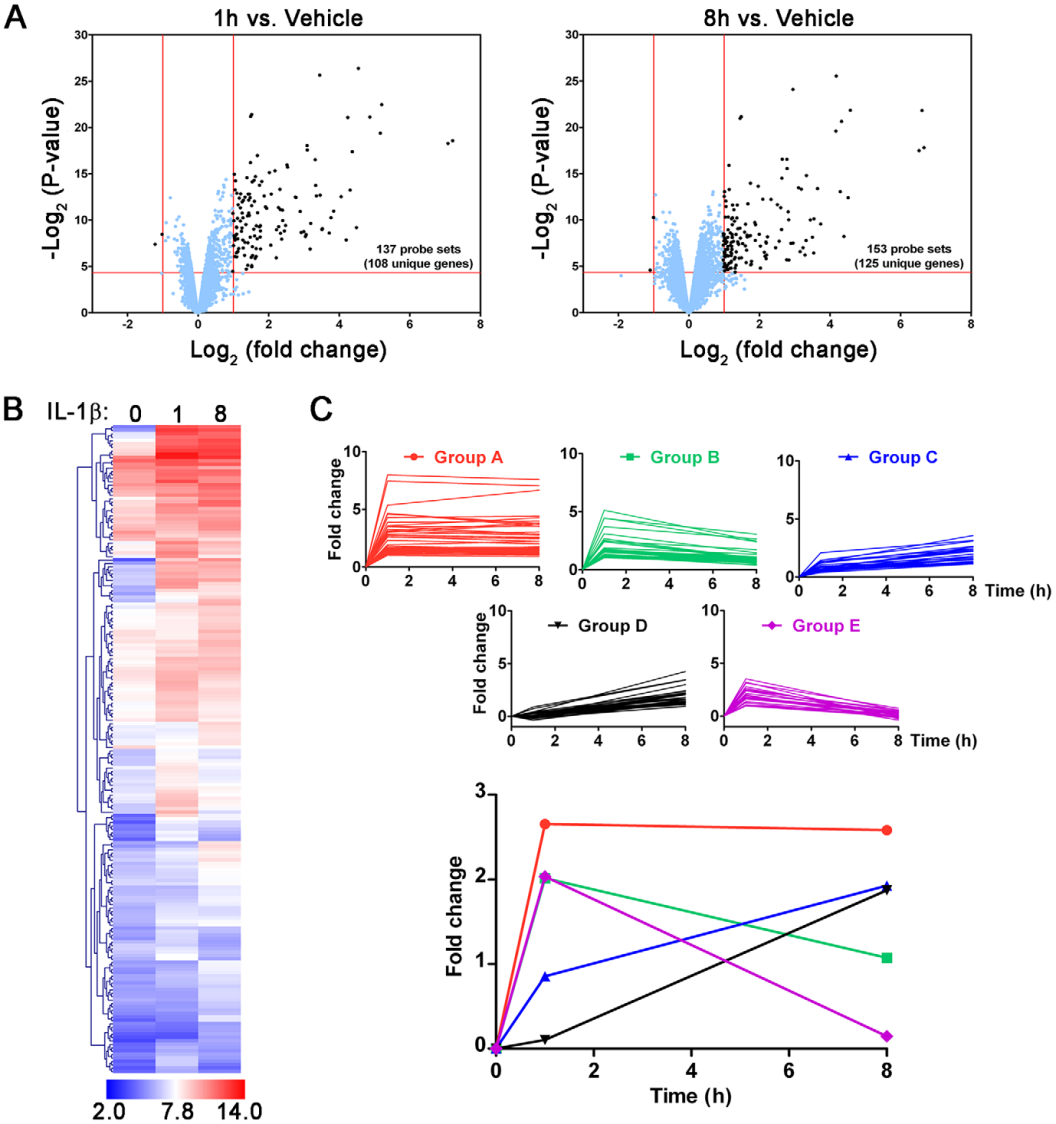
Amnion mesenchymal cell (AMC) microarray data. (A) Volcano plot showing the criteria (±2.0 no-log fold change, <0.05 p-value) for the filtered (blue points) and unfiltered (black points) AMC microarray data. (B) Hierarchical clustering heatmap of discriminant genes from analysis of AMC microarray data (a complete list of the discriminant genes is found in **Table S1**). (C) Top significant profiles from temporal expression analysis of the AMC data; the bottom graph shows the average fold change for each profile. Details of the genes that mapped to each temporal profile are found in **Table S2**.

To expand upon the prior published results [12], we analyzed the expression of 14,500 genes in AMCs in three biological replicates at 1 and 8 h following IL-1β challenge (10 ng/ml) using transcriptional profiling. After background correction, normalization, and filtering of genes with low expression, a 2-fold or greater increase (p<0.05) in transcripts was observed for 137 probe sets (representing 108 unique genes) in AMCs after 1 h of IL-1β stimulation, while 153 probe sets (representing 125 unique genes) were up-regulated by 2-fold or greater after 8 h of cytokine exposure, as summarized in the volcano plots shown in ****Figure 1A****. Compared with the 1 h time point, 48 probe sets (representing 38 unique genes) were significantly (p<0.05) upregulated at 8 h, while 48 probe sets (representing 39 unique genes) were downregulated. The full list of 190 unique probe sets with their average log_2_ expression values at each time point is presented in **Table S1**. The normalized AMC microarray data have been deposited in NCBI's Gene Expression Omnibus [27] and are accessible through GEO Series accession number GSE26315 (http://www.ncbi.nlm.nih.gov/geo/query/acc.cgi?acc=GSE26315).

The 190 unique genes with statistical significance (p<0.05), ≥2 fold-changes in at least one of the pairs of time point comparisons were subjected to hierarchical clustering (**Figure 1B**). There were three major groups into which the genes clustered, each with subgroups bearing differing temporal expression profiles. Genes that exhibited the most dramatic levels of induction following IL-1β challenge included those encoding several cytokines (*Il1a*, *Il6*, *Tnf*, and *Csf2*) and chemokines (*Cxcl1*, *Cxcl2*, *Cxcl3*, *Ccl2*, and *Il8*), the adhesion molecule *Vcam1*, transcription regulatory proteins (*Irf1*, *TnfaipP3*, *Nfkbia*, and *Zc3h12a*), the prostaglandin synthesizing enzyme *Ptgs2*, a stress-activated protein kinase (*Nuak2*), and an apoptotic suppressor protein that interacts with the TNFR2-TRAF signaling complex (*Birc3*). The basal levels of expression (represented as the log_2_ in **Figure 1B**) varied widely, with some genes exhibiting low levels of basal expression (including *Bcl2a1*, *Birc3*, *Ccl5*, *Csf2*, *Cxcl3*, *Icam4*, *Tnf*, and *Vcam1*), and others exhibiting relatively high levels of basal expression (e.g., *Cebpd, Cxcl6, Ier3, Il8, Nfkbia, Plau,* and *Rgs2*).

Due to dramatic differences in the absolute expression intensities, hierarchical cluster analysis *per se* was insufficient to group co-expressed (and presumably, co-regulated) genes based upon relative levels of induction. To evaluate subgroups of genes with similar temporal expression patterns, the 190 unique genes selected above were subjected to clustering using the Short Time-series Expression Miner (STEM) software [31]. Of the 80% of genes that were mapped to temporal expression profiles, five major patterns were discerned (**Figure 1C**): genes that were induced at 1 h for which expression remained elevated (Group A); genes that were induced at 1 h then gradually repressed (Group B); genes for which expression steadily increased throughout treatment (Group C); genes exhibiting delayed induction (Group D); and genes that were rapidly induced, then repressed quickly (Group E). A complete list of the genes mapping to these profiles is provided in **Table S2**.

The genes that fit the Group A expression profile were significantly enriched for gene ontology (GO) terms such as “leukocyte chemotaxis/migration” and “inflammatory response”. Among these genes were a number of highly-upregulated cytokines and chemokines (*Ccl2, Ccl20, Csf1, Cxcl1, Cxcl2, Cxcl3, Cxcr7, Il1a, Il1b, Il6,* and *Il8*), as well as genes involved in NF-κB activation and its regulation (*Nfkb1, Nfkbie, Tnfaip2, Tnfaip3, Tnfaip8, Tnfrsf9,* and *Traf1*). The Group B profile was enriched for the GO term “regulation of gene expression”, and included several genes encoding transcriptional regulators (*Ets1, Fosl1, Jun, Maff, Nfkbia, Nr4a2, Phlda1, Rel, Twist1,* and *Zfp36l1*). Numerous transcription factors (*Bhlhb2, Egr1, Egr2, Egr3, Fos, Fosb, Ier2, Junb,* and *Klf6*) were also present among the genes of Group E, for which the GO term “regulation of RNA metabolic processes” was overrepresented. The Group C profile was enriched for GO terms including “cell adhesion” and “integral to plasma membrane”, and encoded proteins involved in cell adhesion (*Icam4, Cldn1*), integral membrane receptor proteins (*Dram1, Nrp2, Olr1, Rarres1*), and membrane transport proteins (*Slc12a7, Slc2a6, Pdpn*). For Group D genes, the most highly-ranked GO term was “response to virus”; interestingly, several interferon (IFN)-inducible genes were present in this profile (such as myxovirus resistance genes [*Mx1, Mx2*], the gene encoding guanylate binding protein (*Gbp1)*, the single-stranded RNA exonuclease *Isg20*, a retinoic acid-inducible gene I [*Rig-I*]-like receptor involved in the host detection of double-stranded RNA viruses [*Ifih1*], and genes of unknown function [*Ifi35, Ifi44, Ifit3*]), in addition to some cytokines/chemokines exhibiting delayed induction (*Ccl5, Cxcl5, Cxcl6*, and *Tnfsf18*). Insofar as the expression of IFN genes (*Ifna* isoforms, *Ifnb1*, *Ifng*, and *Ifnw1*) themselves remained very low throughout the course of treatment (average log_2_ expression values between 2.3 and 4.1, data not shown), it was unexpected that IFN-regulated genes would be upregulated in response to the inciting stimulus of IL-1β [44], [45]. Moreover, it was interesting to note that in addition to the Mx pathway, components of other IFN-induced antiviral proteins (e.g., OAS3, of 2′-5′-oligoadenylate synthetase system) also had statistically significant induction, but fell short of the chosen fold-change threshold [46]–[48].

**Figure 2:**
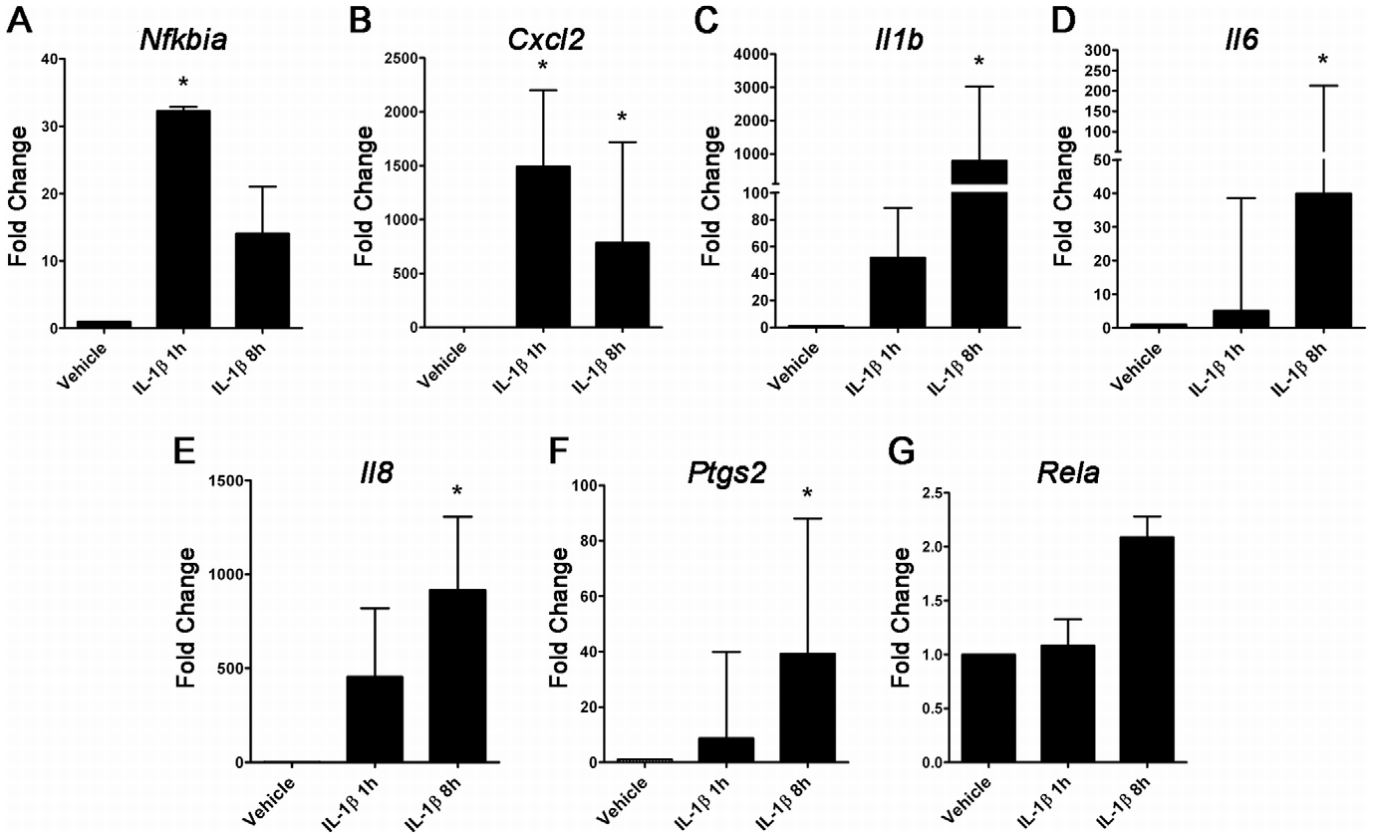
Microarray data verification using qRT-PCR. Fold change of the mRNA expression levels of *Nfkbia*, *Cxcl2*, *Il1b*, *Il6*, *Il8*, *Ptgs2*, and *Rela* in AMC cells treated with 10 ng/ml of IL-1β for 1 h and 8 h and control cells with no IL-1β treatment. Error bars represent standard deviation across at least three independent samples (n = 3 for *Rela* and *Nfkbia*, n = 5 for *Cxcl2*, *Il1b*, *Il6*, *Il8*, and *Ptgs2).*

To verify the results of the microarray analysis, we next examined the expression of seven transcripts (*Rela, Nfkbia, Cxcl2, Il1b, Il6, Il8,* and *Ptgs2*) by qRT-PCR using total RNA collected from replicate experiments using IL-1β treatment conditions identical to those used for the transcription profiling studies. This select mRNA subset was chosen for the frequent high expression of these genes and their cognate proteins in *in vitro* models of inflammation-mediated preterm labor (including the model used in this work) and *in vivo* in biological fluids or tissue specimens collected from women in preterm labor. Consistent with the STEM profile analysis, we found that *Nfkbia* exhibited maximal induction following 1 h of stimulation, with decreased expression thereafter (**Figure 2A**). Of four genes that mapped to the Group A STEM profile (i.e., genes with elevated expression at both 1 and 8 h), only one (*Cxcl2*) followed the predicted temporal course when analyzed using qRT-PCR (**Figure 2B**); the remainder (*Il1b, Il6, Il8*, and *Ptgs2*) exhibited modest upregulation at 1 h, followed by markedly elevated expression at 8 h (**Figure 2, C–F**). These results suggest that, as has been noted in prior studies, microarray analysis tends to underestimate true fold changes in transcript expression [49]. In keeping with this observation, we found that *Rela* expression was moderately increased at 8 h following cytokine challenge by qRT-PCR (**Figure 2G**), but no change was detected by transcriptional profiling.

Collectively, these results confirm that signaling through the IL-1 receptor is biased toward an innate immune response in cultured AMCs. While the inflammatory response became heterogeneous over time due to upregulation of several autocrine/paracrine factors and their receptors, we observed that none of the TLRs surveyed by the microarray platform (*Tlr1* through *Tlr8*) exhibited pronounced levels of expression (average log_2_ expression values between 2.6 and 5.0, data not shown), and therefore were less likely to have contributed substantively to the observed transcriptional response, even under non-sterile conditions. Nevertheless, the IL-1β-induced gene expression changes in AMCs bore some striking similarities to the transcriptional programs resulting from activation of TLR receptors in macrophages [50] and dendritic cells [51]; notably, up to 35% of the IL-1β-responsive genes could also be induced through TLR4. Despite differences in cell type, some degree of overlap in transcriptional response may be expected, inasmuch as TLRs and IL-1Rs both signal via myeloid differentiation factor 88 (MyD88)-dependent pathways, which utilize a common pool of intercessor proteins (such as the IRAKs [IL-1 receptor-associated kinases]) as well as common downstream cascades (e.g., NF-κB, p38 and c-Jun N-terminal kinases [JNKs]) to regulate programs of inflammatory gene expression [38], [52]. Even with these commonalities, TLR and IL-1R signaling diverge in characteristic ways, as is highlighted by the MyD88-independent induction of IFN genes by TLR3 and TLR4 [37], [52], [53]. Consistently, interferon induction was conspicuously absent in our model, despite upregulation of certain IFN-inducible genes (e.g., *Mx1*, *Ifih1* [*Mda5*]).

### Analysis of IL-1β mediated gene expression patterns in AMCs reveals enrichment for inflammatory transcription factor binding motifs

The acute and delayed responses to IL-1β challenge in AMCs are governed by multiple transcription factors with dynamic and combinatorial interactions. The most dominant among these signaling pathways is the NF-κB system [54], the activation of which we have previously characterized in AMCs in response to IL-1β [12], [55]. While NF-κB is a critical regulator of immunomodulatory gene expression, IL-1β expression can, directly or indirectly, influence the activation of a number of pro-inflammatory signaling cascades in parallel, including Janus kinase (JAK), signal transducer and activator of transcription (STAT), and mitogen-activated protein kinase (MAPK) pathways. For example, in WISH cells, which bear many of the same molecular responses to cytokine stimulation as AMCs, we found that IL-1β challenge acutely activates both the NF-κB and MAPK cascades, the latter including pathways employing extracellular signal-regulated kinases (ERKs), c-Jun N-terminal kinases (JNKs), and p38 isoforms [56].

To begin to decipher the full spectrum of potential transcriptional regulatory networks governing IL-1β-induced gene expression in AMCs, we used the Pscan software tool [33], which performs *in silico* computational analysis of overrepresented *cis*-regulatory elements within the 5′-promoter regions of coordinately regulated genes. When Pscan was applied to all 169 genes significantly upregulated in response to IL-1β at either 1 h or 8 h, consensus DNA sequences for NF-κB transcription factors, as well as those for TATA binding protein (TBP) and specificity protein 1 (SP1), were significantly enriched (**Table S3**). Similar results were obtained when the genes were subdivided into subsets based on timing of induction (i.e., those upregulated following 1 h of stimulation, and those for which transcription was induced at the 8 h time point) (**Table S3**). Since many of the IL-1β-induced genes were known to contain NF-κB response elements (κBREs), we next sought to explore conserved regulatory motifs among those genes not known to be regulated by NF-κB. To this end, we compared IL-1β-induced genes in our dataset to a list of 306 known or predicted NF-κB responsive genes compiled from several sources [32], [33], [50], [57], [58] (accessed April 2010). We found that 62/190 (33%) of the induced genes were expected to be NF-κB responsive, suggesting that as much as two-thirds of the remaining genes might be regulated by parallel, IL-1β-activated, but NF-κB independent signaling pathways. When the predicted NF-κB-responsive genes were excluded from consideration, the remaining promoters were most highly enriched for SP1, serum response factor (SRF), TBP, and CCCTC-binding factor (CTCF) binding motifs (**Table S4**). Sixty nine of these genes had perfect or near-perfect binding sequences for SP1, whereas only a few (*Dusp5, Egr1, Egr2, Egr3*, and *Fosl1*) had motifs similar to the consensus sequence for SRF. In addition, some of these remaining genes were enriched for κBRE-like motifs, and further analysis of these latter sequences revealed an additional 26 candidate target genes for NF-κB (*Bdkrb2, Bhlhe40, Btg2, Ccnl1, Cxcr7, Dram1, Dusp2, Egr1, Egr2, Gfpt2, Hes1, IFIH1, ISG20, Jun, Klf6, Nab1, Ninj1, Nr4a2, Ppp1r15a, Ptger4, Ripk2, Rrad, Sat1, Slc12a7, Socs3,* and *Zfp36*).

Despite the transactivation of numerous NF-κB responsive genes, it remained that only 20% of the 306 genes predicted to be NF-κB responsive [32], [33], [50], [57], [58] (accessed April 2010) were significantly induced in AMCs following IL-1β challenge. Among potential reasons for limited induction of the regulation in this model, we considered that transrepression by nuclear receptors, which are known to inhibit overlapping but distinct subsets of NF-κB responsive genes [59], might have contributed. Surprisingly, upon review of the normalized, unfiltered microarray data, we found that cultured AMCs exhibited relatively low levels of expression of transcripts for numerous nuclear receptors, including progesterone and estrogen receptor isoforms, and several isoforms of the peroxisome proliferator-activated receptor (PPAR) family (**Table S5**). Of 48 nuclear receptors surveyed, only eleven exhibited relatively high (log_2_ expression ≥6) transcript abundance: Retinoic acid receptor alpha (*Rara*), nuclear receptor subfamily 2, group F, member 6 (*Nr2f6*), estrogen-related receptor alpha (*Esrra*), retinoid X receptor alpha and beta (*Rxra, Rxrb*), Rev-ErbA alpha and beta (*Nr1d1, Nr1d2*), liver X receptor beta (*Nr1h2*), COUP 1 and 2 transcription factors (*Nr2f1, Nr2f2*), and the glucocorticoid receptor (*Nr3c1*). At the protein level, we confirmed that progesterone receptors and PPAR-Y exhibited low expression levels in AMCs using immunoblotting (data not shown). Although *Nr4a2* (nuclear receptor subfamily 4, group A, member 2, also known as *Nurr1*) was significantly upregulated following IL-1β stimulation, this transcript exhibited relatively low transcript levels following induction. These data suggest that the potential for nuclear receptor cross-talk in AMCs may be significantly more restricted in comparison with other cell types comprising the fetal membranes.

To confirm the role of NF-κB in the transactivation of a subset of inflammatory genes, we utilized the 26 *S* proteasome inhibitor MG-132 (N-acetyl leucinyl-leucinyl-leucinal), which prevents translocation of NF-κB from the cytoplasm to the nucleus [12], [56]. When AMCs were preincubated with MG-132 (30 μM) before IL-1β stimulation for 1 h, the expression of transcripts encoding *Cxcl2, Il1b, Il6,* and *Il8* were significantly diminished (**Figure S1**). Marked attenuation of the mRNAs encoding these cytokines and chemokines was noted at 8 h. The effect of MG-132 in IL-1β-induced *Ptgs2* expression was almost identical to that we have previously characterized in a prior report [12], in that MG-132 only partially inhibited cytokine-induced upregulation. Collectively, these results support a role for NF-κB as a master regulator of both acute and delayed responses in this subset of genes.

**Figure 3:**
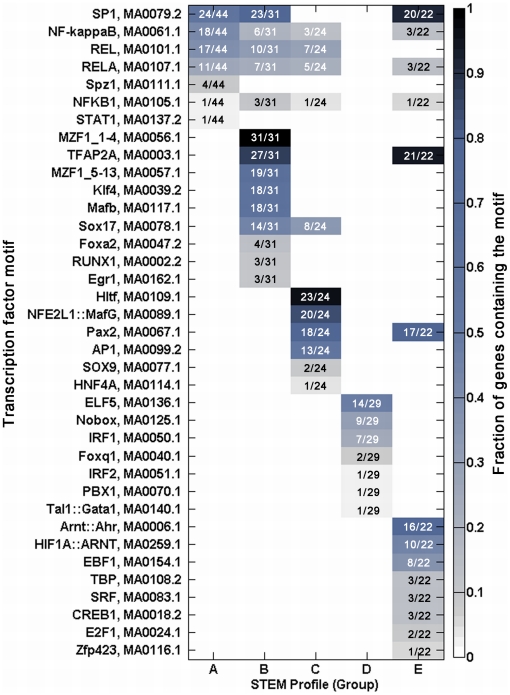
Patterns of transcription factor binding motif enrichments within promoters of genes from each temporal expression profile of the AMC data. Each column in the matrix represents a temporal expression profile, and each row represents a transcription factor binding element. Each profile (column) corresponds to those in Figure 1C, and each colored block in the matrix indicates a pair of motif and temporal expression profile for which a fraction (indicated in the blocks) of the genes in the profile is enriched for the motif (Pscan score of ≥0.95). The color of the blocks corresponds to the fraction of genes in the profile enriched for each transcription factor motif, and corresponds with the color scale shown on the right.

To further delineate conserved transcription factor binding motifs among coordinately regulated genes, we next subdivided the genes based on the temporal expression clusters revealed by STEM analysis (**Table S2**). In general, these could be divided into genes for which expression was rapidly induced (Groups A, B, and E), and genes exhibiting delayed induction (Groups C and D). When considered together, the genes acutely responsive to IL-1β (Groups A, B, and E) were highly enriched for potential SP1 and NF-κB response elements. The genes of Group C were also enriched for NF-κB binding motifs, in addition to those for activator protein 1 (AP1). By contrast, the response elements most highly enriched in the promoter regions of Group D genes were interferon-stimulated responsive elements (ISREs), specifically, interferon regulatory factors 1 and 2 (IRF1, IRF2). To refine this model, we next examined the specificity of the match between each motif (represented as a position specific weight matrix) and target sequences within individual gene promoters (**Figure 3**). In so doing, we found that certain overrepresented motifs did not have well-matched oligonucleotide sequences within any promoter (defined by a score of ≥0.95 based on a relative scale from 0 to 1, in which “1” corresponds to a sequence having the best match when compared to a given position specific weight matrix [33]), and such motifs were omitted from further consideration. Of the motifs with at least one well-matched predicted binding sequence, four (SP1, NF-kappaB, RELA, and NFKB1) remained commonly represented in the acute response genes (Groups A, B, E), and the three NF-κB binding motifs (NF-kappaB, RELA, and NFKB1) were also found in the promoters of Group C genes (**Figure 3**). In addition, we observed that Groups B-E exhibited some mutually-exclusive patterns of motif enrichment (note the staggered arrangement of enriched motifs in **Figure 3**), including Group D for which all putative regulatory sequences were discrete from the remaining profiles.

**Figure 4:**
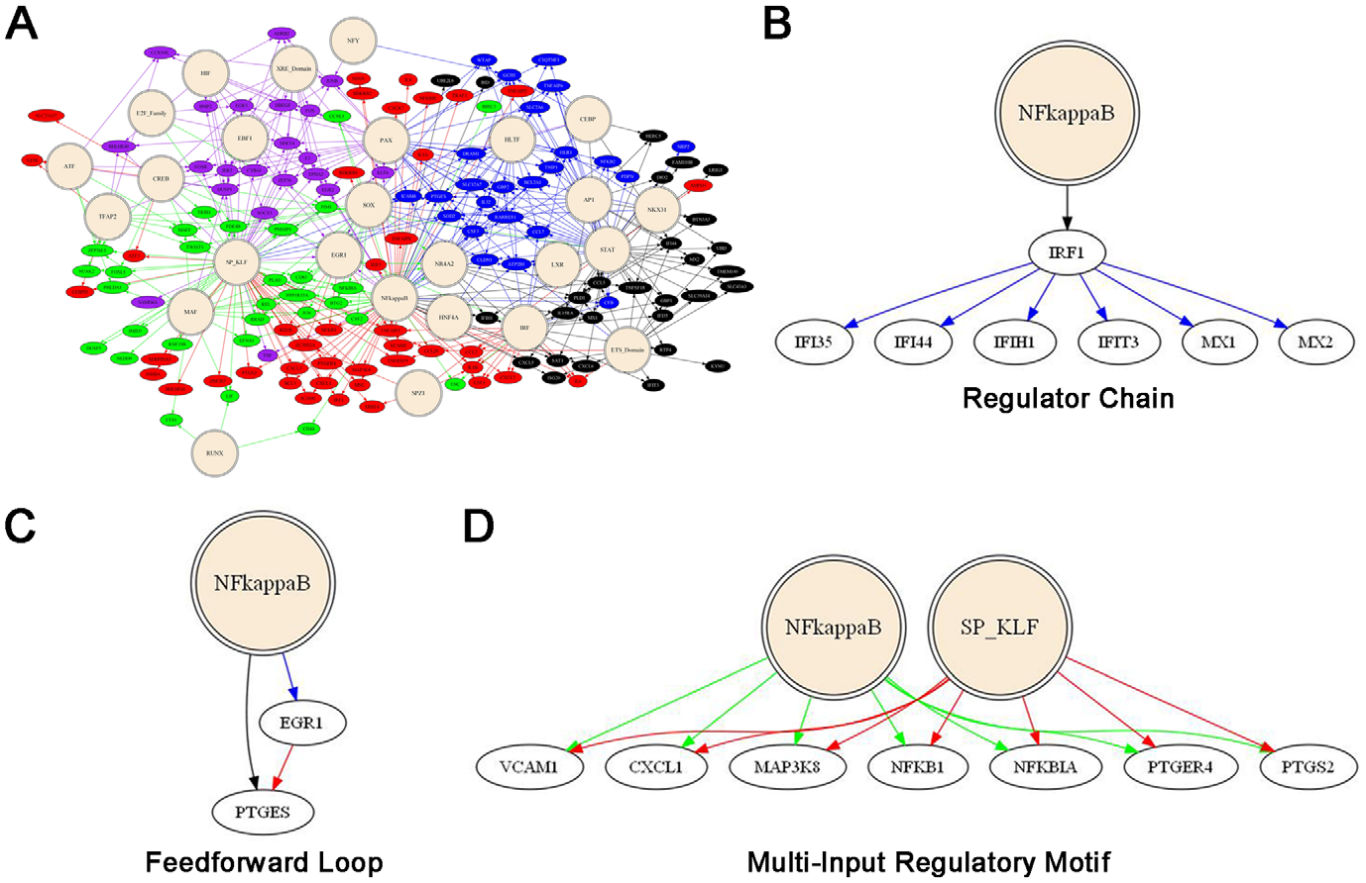
Gene regulatory network of AMC dataset inferred from transcription factor binding motif results. (A) Overview of gene regulatory network. Double circles represent binding motifs and ovals represent genes. Lines between motifs and genes represent inferred regulation based on Pscan motif analysis. The genes and respective connecting lines are colored based on the STEM profile groups depicted in Figure 1D (group A = red, group B = green, group C = blue, group D = black, group E = purple). (B–D) Types of regulatory subnetworks represented.

By combining the expression data (clustered by relative temporal expression pattern) with the results of the motif enrichment analysis, we next constructed a potential gene regulatory network for IL-1β-stimulated AMCs (**Figure 4A** and **Figure S2**). Although the Pscan data suggested interactions between specific transcription factors and target genes, in reality, the DNA sequences giving rise to such predictions may be bound by multiple proteins, which are typically members of a homologous protein family [60]. For instance, the SP1 regulatory element (GC box) may be used by several members of the Sp/Krüppel-like factor (KLF) transcription factor family and as such, enrichment alone does not necessarily specify which factors may be utilized [61]; indeed, some Sp/KLF factors bind to identical motifs (e.g., SP1 may compete with SP3, KLF4, KLF9, and/or KLF13 in the context of certain promoters). To account for such redundancies, transcription factors in our model were cast as categorical nodes (tan circles in **Figure 4A** and **Figure S2**) based on homologous binding activity to specific regulatory elements, with direct connections to target genes predicted by promoter scanning (these nodes and edges were color-coded based on the STEM expression profiles depicted in **Figure 1C**). As an example of such consolidation, promoter scanning revealed that two domains of myeloid zinc finger gene 1 (*Mzf1*, consisting of zinc fingers 1 to 4 and 5 to 13), were overrepresented in certain promoters; however, since these regulatory elements were highly similar to κBREs [62], the two MZF1 motifs were modeled only as NF-κB motifs, which were more relevant given the chosen perturbation. From the network model, it was apparent that the late-response genes (Groups C and D, represented as blue and black nodes/edges, respectively) clustered together, and exhibited a high degree of connectivity with transcriptional regulators distinct from those during the acute response to IL-1β challenge (**Figure 4A** and **Figure S2**).

Although not unequivocal, the results of this *in silico* analysis offer important clues to potential regulatory networks activated through the IL-1 receptor. From a global perspective, many of the genes examined, particularly those which displayed superactivation between the 1 and 8 h time points (e.g., genes in the Group A STEM profile), could be considered as multi-input regulatory motifs (**Figure 4D**) [63], in which several transcription factors contribute in a combinatorial manner to the observed transcriptional response. Of the genes significantly upregulated following to IL-1β challenge, κBRE *cis*-acting elements were present in about half (89/188) of the genes, with co-enrichment for SP1, TFAP2A (transcription factor activating enhancer protein-2 alpha), SOX (Sry-related HMG box), and STAT response sequences. The promoters for the remaining 99 genes without apparent κBREs (at least within the upstream regulatory regions) were enriched for Sp/KLF, Forkhead box (FOX), and IRF transcription factor binding motifs, among others. Some inferences about the transcriptional control of late-response genes could also be made. For example, since several AP1 family members (*Fos*, *Fosb*, *Jun*, *Junb* and *Atf3*) were significantly induced during the acute response to IL-1β, these transcription factors could have contributed to the delayed transcriptional response, especially among the genes of Group C (**Figure 3** and **Figure S2)**. As well, although IFN genes were not induced, that for IRF1 (interferon regulatory factor 1) was rapidly upregulated in response to IL-1β. As such, the delayed induction of certain components of the IFN-response circuit (enriched in the Group D STEM profile) could have been regulated indirectly through IRF1 [46] as part of a regulator chain subnetwork (in which an initiating factor promotes the expression of a second factor, which in turn regulates a subset of genes, **Figure 4B**) [63]. In addition, the gene for the early growth response-1 protein (*Egr1*) was transiently induced at 1 h, which could have contributed to the induction of several late-response genes, such as *Ptges* [64], [65], in conjunction with NF-κB [12]. Such a subnetwork is reminiscent of a feedforward regulatory loop, in which a single factor promotes the expression of both a target and an enhancer of that target (**Figure 4C**) [63]. Finally, both *Klf6* and *Klf9* were significantly upregulated during the acute response to IL-1β, which could have contributed to the regulation of certain genes (e.g., *Icam4* and *Sod2*) exhibiting delayed activation. Other transcription factor motifs of note included TFAP2A (AP-2α) [66], which has a significant number of well-matched elements among the promoters of genes in STEM profiles B and E (**Figure 3**). AP-2α is transcription factor with context-dependent activator and repressor functions, and is important in cellular morphogenesis [67]–[69] by binding to variations of the GC-rich sequence 5′-(GCCN)_3_GGC-3′ [68]. The top represented motifs for STEM profile C were helicase-like transcription factor (HLTF) and nuclear factor (erythroid-derived 2)-like 1 (NFE2L1). Helicase-like transcription factor is a member of the SWItch/Sucrose NonFermentable (SWI/SNF) family of chromatin remodeling enzymes, and may play a role in error-free DNA replication [70], [71]. The NFE2L1 motif represents the binding motif of heterodimer transcription factor 11 (TCF11) with the small MafG protein: 5′-TGCTgaGTCAT-3′, so named because the binding-site is identical to the NF-E2-site. Aside from interacting with MafG proteins, TCF11 binds to a subclass AP1 sites [72].

### Transcriptional regulatory networks in full-thickness fetal membranes in the setting of term labor

Having established a paradigm to decipher potential transcriptional regulatory networks in a dynamic *in vitro* system, we next applied this strategy to clinical specimens. For this, data were abstracted from a transcriptional profiling study [19] conducted using samples of full-thickness fetal membranes (amniochorion and maternal decidua) from patients delivered at term in the absence or presence of spontaneous labor (N = 12 per group). In this cross-sectional study, all specimens were subjected to pathological examination, and no specimens with histologic chorioamnionitis were included in the analysis. The dataset included 224 discrete probe sets corresponding to 197 unique transcripts which were queried using the HG-U133A and HG-U133B arrays (Affymetrix).

**Figure 5:**
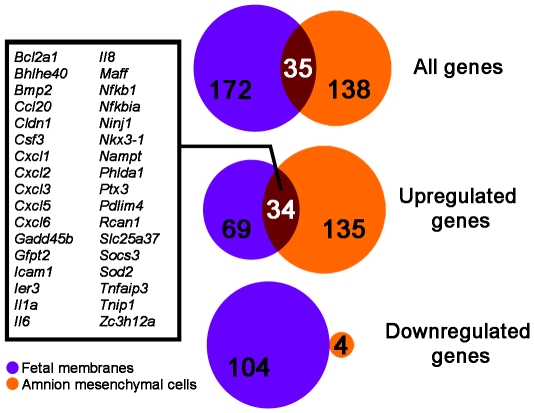
Venn diagram of comparing the genes of the AMC and Haddad *et al* datasets. The Venn diagrams depict differentially expressed genes from the Haddad *et al.* data obtained from fetal membranes following term labor, and the genes significantly expressed in AMCs at 1 h and 8 h post IL-1β treatment when compared to control. The diagrams depict the amount of overlap of all the genes, just the upregulated, and the downregulated genes (top to bottom). The inset box lists the 34 genes that are common and upregulated in both the fetal membranes and AMC data. An additional gene, type II iodothyronine deiodinase (*Dio2*), was common but divergent between the two datasets in that it was downregulated in the fetal membranes while upregulated in AMCs.

**Figure 6:**
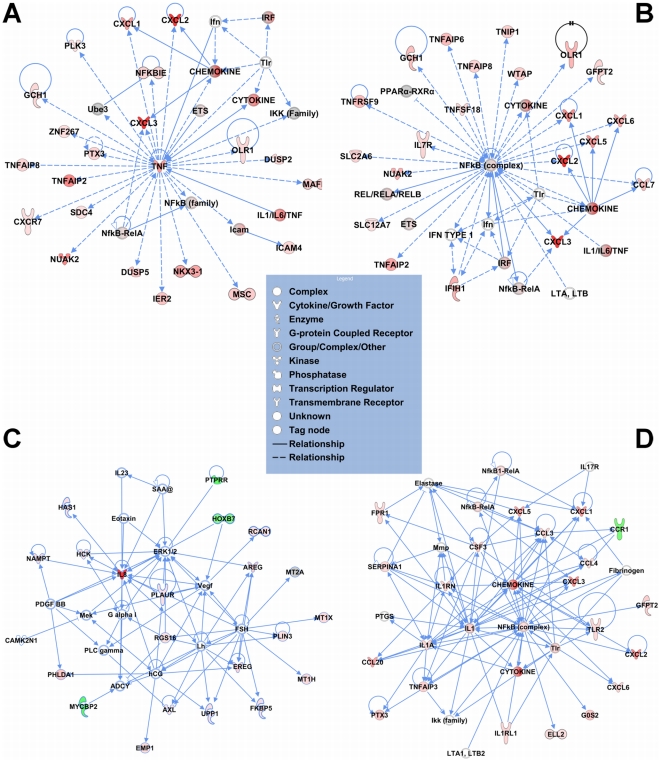
Top networks for each time point (1 h and 8 h) in the AMC microarray data and for the Haddad *et al.* TIL versus TNL microarray data. (A) The top network of 1h IL-1β treatment of ACC with a score of 44, showing a prominence of TNF and NF-κB family. (B) The top network of 8 h post IL-1β-treatment of ACC with a score of 41. This network shows high connectivity of the relevant genes with the NF-κB family. (C) The top network of the Haddad dataset with a score of 35, showing hormones (FSH, LH, hCG) and growth factors (AREG, EREG, PDGF, VEGF). (D) The second ranking network of the Haddad dataset with a score of 33, showing an inflammatory signature. In the networks, molecules are represented as nodes, and the biological relationship between two nodes is represented as an edge (line). The intensity of the node color indicates the degree of up- (red) or down- (green) regulation. Nodes are displayed using various shapes that represent the functional class of the gene product, as explained in the legend.

To assess how faithfully our simplified *in vitro* model recapitulated the tissue-level data, we compared the gene expression profile in IL-1β-stimulated AMCs with that of Haddad *et al.* [19]. There were 41 unique probe sets (35 genes) in common (**Figure 5**), and of these, 34 genes were upregulated in both datasets, whereas no common downregulated genes were identified. The genes in common included several cytokines and chemokines that were identified previously as part of the “acute inflammation gene expression signature” following spontaneous labor onset [19]. One gene, type II iodothyronine deiodinase (*Dio2*), was divergent between the two datasets in that it was downregulated in Haddad *et al.* while upregulated in AMC. To further compare the AMC and Haddad *et al.* datasets, both were subjected to pathway analysis using Ingenuity Pathway Analysis Software (IPA, Ingenuity Systems, www.ingenuity.com) under identical conditions of log_2_-fold change cutoff of 1.0 (corresponding to a fold change of 2), and averaging of multiple probesets that correspond to the same gene. In congruence with the other analyses , an inflammatory signature featuring NF-κB was prominent in the top ranking interaction networks at both 1 h and 8 h following IL-1β treatment in the AMCs when compared with the vehicle control (**Figure 6, A and B**, with scores of 44 and 41, respectively). The two top scoring interaction networks (scores of 35 and 33, respectively) from the Haddad *et al.* dataset are shown in **Figure 6, C and D**. Not surprisingly, the common molecules among the top networks from the AMC dataset and the second-ranked network from the Haddad *et al*. dataset were molecules related to inflammation (*i.e*., chemokines, pro-inflammatory cytokines, leukotriene synthesizing enzymes, members of the NF-κB family, *Tlr2*, etc.). Interestingly, the top ranking interaction network from the Haddad *et al.* dataset contained hormones (follicle-stimulating hormone [FSH], luteinizing hormone [LH], human chorionic gonadotropin [hCG]) and growth factors (epidermal growth factor receptor ligands amphiregulin [AREG] and epiregulin [EREG], platelet-derived growth factor [PDGF], and vascular endothelial growth factor [VEGF]). While the specific relevance of these molecules within the fetal membranes may be limited at this time, that such a network was so highly-ranked in the clinical specimens, but was not present in the IL-1β-stimulated AMCs, suggests that the level of complexity among interactions *in vivo* (i.e., multiple cell type interactions) was far greater than in the simplified *in vitro* model. A list of all the networks from the AMC and fetal membranes [19] along with their scores and molecules is found in **Tables S6, S7, and S8**. Pathway and biological function analysis using IPA provided further support for an inflammatory signature, as interleukin and immune cell signaling pathways were among the top ten statistically significant canonical pathways, while inflammatory response was the second most significant biological function. Plots of the top ten canonical pathways and biological functions for the AMC and Haddad *et al*. datasets and their Benjamini Hochberg p-values are found in **Figure S3**.

**Figure 7:**
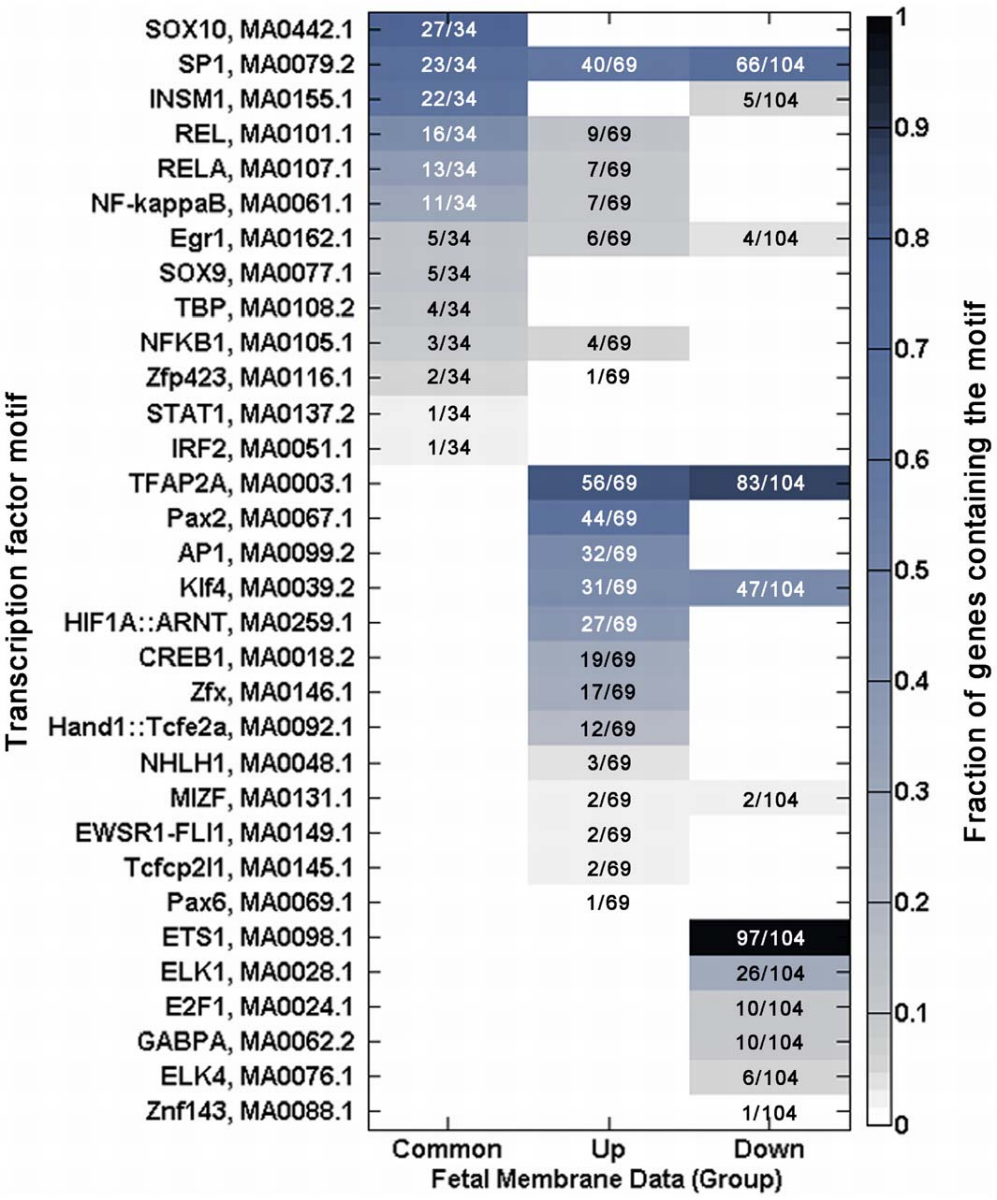
Patterns of transcription factor motif enrichments within promoters of genes from the Haddad *et al.* dataset. Each column in the matrix represents three clusters of genes: the common upregulated genes between the Haddad *et al.* and AMC datasets, and the genes uniquely upregulated and downregulated in the Haddad dataset. Each row represents a transcription factor binding element. Each profile (column) corresponds to those in Figure 1C, and each colored block in the matrix indicates a pair of motif and gene cluster for which a fraction (indicated in the blocks) of the genes in the profile is enriched for the motif (Pscan score of ≥0.95). The color of the blocks correspond to the fraction of genes in the profile enriched for each transcription factor binding motif, and corresponds with the color scale shown on the right.

**Figure 8:**
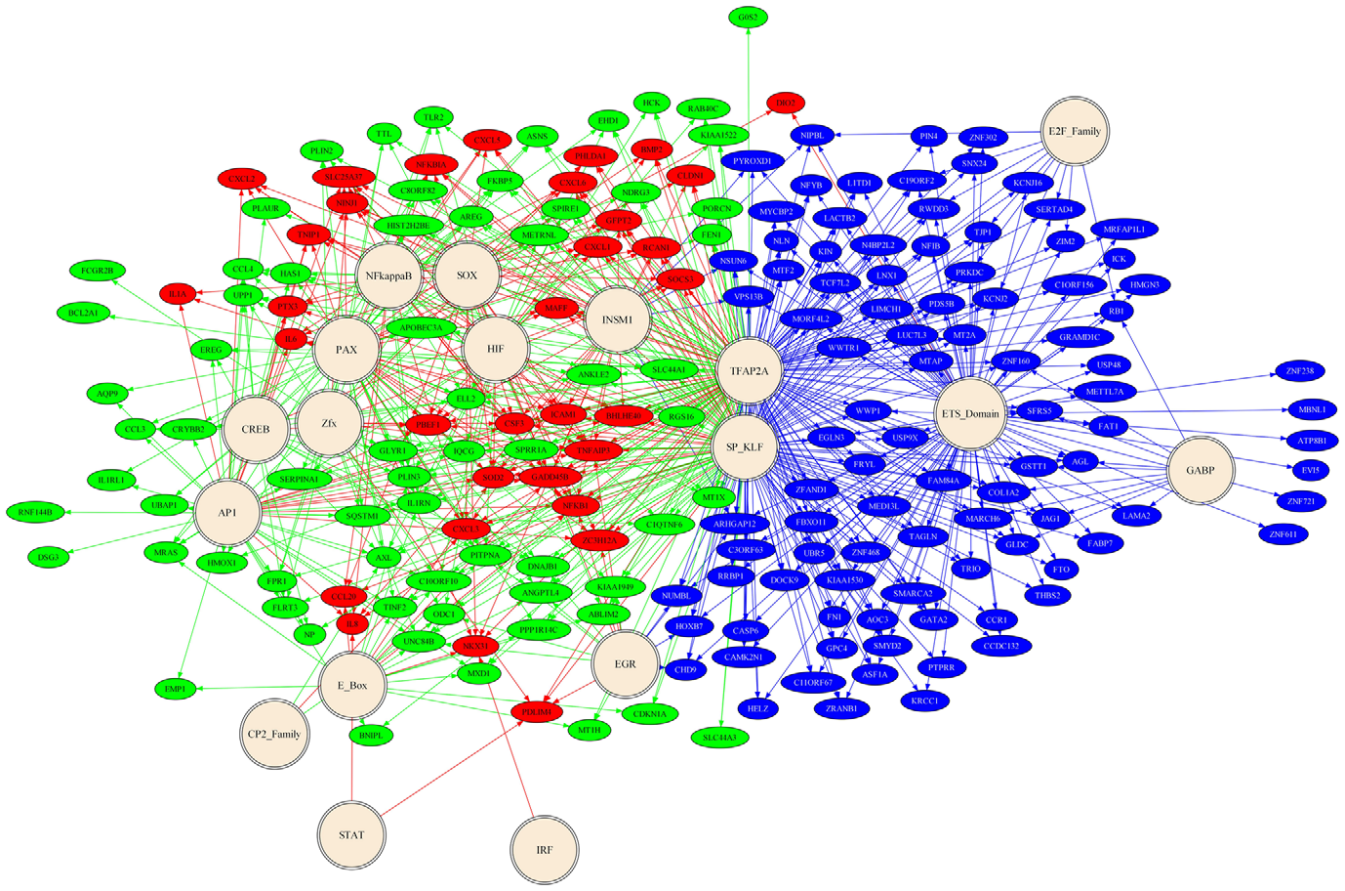
Gene regulatory network of the Haddad *et al*. dataset inferred from transcription factor binding motif results. Double circles represent binding motifs and ovals represent genes. Lines between motifs and genes represent inferred regulation based on Pscan motif analysis. The genes and respective connecting lines are colored based on the grouping in **Figure 7**: common genes with AMC dataset (red), upregulated genes (green), and downregulated genes (blue).

Of the genes upregulated in fetal membranes in the setting of labor, 33% were also induced following IL-1β treatment in AMCs (**Figure 5**). To further examine putative regulatory pathways activated in the fetal membrane specimens, motif enrichment analysis for differentially expressed genes was performed using Pscan [33]. For this, genes were subdivided into three groups: those which were upregulated in both fetal membranes and AMCs (the “core inflammatory” subset), those which were upregulated in fetal membranes only, and those which were downregulated in fetal membranes (**Figure 7**). This information was then used to construct a gene regulatory network model (**Figure 8**). As anticipated, the promoters of core inflammatory genes were enriched for κBREs as well as Sp/KLF binding motifs. The remaining transcription factor binding motifs enriched in this subset were present in both the tissue samples and the cytokine-stimulated AMCs, with the exception of that for insulinoma-associated protein 1 (INSM1). SOX consensus sequences (SOX9 and SOX10) were more highly enriched among the core inflammatory subcluster than in IL-1β-induced genes in AMCs. While the relevance of this finding *in vitro* is uncertain, it is plausible that mechanical signals, such as those produced in active term labor (e.g., uterine stretch, cervical dilatation), might induce the expression of *Sox* transcription factors *in vivo*. An association between mechanotransduction and *Sox* gene expression has been demonstrated in chondrocyte models [73]. Although *Sox* transcription factors were not differentially regulated in following labor in the fetal membrane dataset, it is interesting to note that the expression of *Sox18* was identified among a group of labor-associated genes in term myometrium [74]. Of motifs enriched among the promoters of genes upregulated solely in the fetal membrane dataset, those for Sp/KLF, TFAP2, paired box (PAX), AP1, hypoxia-inducible factor (HIF), and zinc finger X-chromosomal protein (Zfx) had the greatest number of well-matched predicted binding sequences. A small portion (13%) of genes in this subset also contained NF-κB response elements.

Despite divergent gene expression profiles, the predicted gene regulatory network in fetal membranes exhibited a great degree of overlap in motif enrichment (**Figures 7** **,** **8**) with the AMC network (**Figure 3** and **Figure S2**), suggesting the use of common global transcriptional networks. It is noteworthy that 39% of the genes upregulated solely in the Haddad *et al.* dataset had promoters with HIF response elements, which is relevant given that uterine contractions during labor cause intermittent utero-placental hypoperfusion [75], [76]. Indeed, a recent study by Cindrova-Davies and colleagues found that term labor was associated with stabilization of hypoxia-inducible factor-1α and changes in the expression of transcripts and proteins associated with oxidative stress and angiogenic regulation in placental tissue [77]. Aside from genes potentially regulated by HIF isoforms, hypoxia-reoxygenation may also be reflected in inflammatory gene induction following labor, since oxidative stress itself is associated the activation of several inflammatory signaling cascades (e.g., *Mapk* and NF-κB) [78], [79]. Collectively, in addition to inflammation, these results suggest that fetal membranes exposed to labor possess global gene expression changes associated with hypoxia-reperfusion.

## Discussion

This study describes a computational approach based on transcriptional profiling for predicting global gene regulatory networks (GRNs) invoked in the propagation of inflammatory responses. As with prior work [45],[50],[57],[80]–[82], the methodology outlined here strategically combines methods for identifying clusters of coexpressed genes with an analysis of transcriptional regulatory elements enriched in the promoters of these genes. While the present analysis is by no means exhaustive, it offers proof-of-principle for the application of GRN modeling to the study of human parturition. Importantly, this approach complements the pathway and gene-interaction networks analyses commonly employed in the analysis of high-dimensional datasets, and offers further insight into the transcriptional control of labor. Clearly, more comprehensive, reductionist, and mechanistic approaches are required to demonstrate the rigorous authenticity of these global gene expression observations.

Many of the previous transcriptional profiling studies using intrauterine tissues [19], [20], [74], [83]–[87] have relied on pathway analysis to discern overrepresented functional classifications that identify potential gene-interaction networks. While these methods are informative, analysis of enriched or impacted pathways has several inherent limitations. These include: (1) ambiguity in pathway classification (non-standardized categorization of biological pathways leads to groupings that are subjectively defined); (2) software-dependent differences in the generation of specific interaction networks, depending upon how gene-gene (intermodal) relationships are defined, and what resources are used to define such interactions [88]; (3) overrepresentation of well-studied pathways, to the exclusion of potentially important genes for which little experimental data is available; and (4) bias toward interaction networks having larger numbers of genes [89]. Furthermore, in addition to coexpressed genes, groups of randomly selected genes will also result in significantly overrepresented interaction networks [89]. Finally, interaction networks are theoretically based, and may have little physiological relevance within a given biological system [88].

In light of these considerations, we focused on deciphering potential transcriptional regulatory programs that may account for specific gene expression signatures that differentiate labored from unlabored intrauterine tissues. Many bioinformatic approaches currently exist to aid in the reconstruction of GRNs [90]–[93]. Generally speaking, GRNs are reverse-engineered from transcriptional profiling data, based on the assumption that coordinately expressed genes are likely to be controlled by a common group of transcription factors [94]–[97]. In most instances, genes from transcriptional profiling experiments must first be grouped into units of coexpressed genes. Numerous computational algorithms have been developed to partition such coexpression clusters [91], [98]. These approaches utilize differing theoretical frameworks for data partitioning, including clustering algorithms (e.g., hierarchical or partitional clustering), probabilistic graphical models [99], [100], matrix decomposition approaches [101]–[105], and algorithms that incorporate multiple lines of experimental evidence [106], [107]. The specific usefulness of such algorithms depends upon the intended use, as well as the benefits and limitations of these methods are reviewed elsewhere [90], [93], [98].

In developing the GRN models presented here, we first utilized AMCs as simplified *in vitro* model system, for which temporal gene expression data could be generated. This approach enabled comparison of different methods for partitioning the microarray data prior to motif enrichment analysis. We found that clustering based upon relative transcript expression (STEM analysis [31]) was more informative than hierarchical clustering, inasmuch as that the latter was biased toward segregation based on absolute expression values. Using hierarchical clustering, we found that many genes exhibiting similar patterns of temporal activation remained ungrouped (**Figure 1B**), which confounded the accurate determination of common gene regulatory mechanisms during subsequent analysis. By considering longitudinal relative expression patterns, we distinguished regulatory mechanisms governing genes with rapid transcriptional activation from those controlling genes for which expression was delayed. These alterations in transcriptional regulatory mechanisms likely reflect the cumulative influence of cytokines, chemokines, eicosanoids, and other signaling molecules which develop over time. This analysis revealed dynamic interplay between network elements, as illustrated in the topology models (**Figure 4**). For example, early upregulation of genes encoding key transcription factors (such as *Irf1* and *Egr1*) could be linked plausibly to the regulation of genes exhibiting delayed induction. Although such inferences require that transcript abundance be correlated with regulatory activity [97], this analysis suggests network structure that can now be tested experimentally.

Once genes are partitioned into suitable modules, the core promoters of coexpressed genes (typically, regulatory regions within −1000 to +50 bp relative to the transcriptional start site) may be evaluated for overrepresented *cis*-regulatory elements [96]. Of the two ranges available in Pscan that are closest to this region of interest (−950 to 50 and −1000 to 0), the −950 to +50 bp range was selected for the analyses. A host of computational algorithms for motif enrichment analysis currently exist (reviewed in [96], [108]–[111]), including OTFBS [112], YMF [113], CLOVER [114], CARRIE [94], oPOSSUM [115], MAPPER [116], CORE_TF [117], TFM-Explorer [118], PAP [119],and PASTAA [120], among others. The Pscan algorithm [33] applied here utilizes computational identification of known transcription factor binding sites using position specific weight matrices; however, the identification of enriched short DNA sequences without bias (*ab initio* prediction) is also possible [80], [109], [121], [122]. The utility of these algorithms varies, depending upon the model organism under investigation (performance is typically best when applied to lower organisms with a more limited repertoire of transcription factors [111]). In addition, the specific means used for identifying sequences (e.g., position weight matrices, Markov models, Bayesian trees, and variable order models [123]), and the statistical methods used to determine the degree to which identified sequences are overrepresented [109] also influences the usefulness of these *in silico* methods. While several of the web-based computational algorithms for motif enrichment analysis were surveyed in the current study (data not shown), we chose not to perform an exhaustive comparison of these tools in this work, particularly given the rapid rate at which novel programs become available; rather, we chose to focus on the general approach as proof-of-principle, which can be modified and adapted using existing datasets.

An advantage of motif enrichment analysis is that it facilitates the distinction between primary and secondary targets of a given transcription factor, since primary targets are more likely to contain *cis*-regulatory elements for that factor [90], [124]. However, computational analysis of transcription factors in isolation may be insufficient to elucidate combinatorial interactions that may ultimately govern the transcriptional response of a particular gene. For example, our analysis of cytokine-stimulated AMCs revealed candidate primary NF-κB response genes exhibiting dramatic differences in activation kinetics, suggesting that target genes with delayed activation (*Ccl5, Ccl7, Cfb, Cxcl5, Cxcl6, Dram1, Gch1, Ifih1, Il15ra, Il32, Isg20, Mx2, Nfkb2,* and *Tnip1*) require regulatory mechanisms distinct from those governing early-response genes. Recent data now suggest that rapidly-activated NF-κB responsive genes (e.g., *Il6*, *Ccl20*, and *Icam1*) respond in a graded (analog) fashion, whereas certain late NF-κB response genes (e.g., *Ccl5*) are upregulated only above a certain activation threshold (digital transcriptional regulation) [125], [126]. While the mechanistic basis for such observations may depend on epigenetic modifications affecting chromatin configuration, it is possible that cooperative interplay between NF-κB and other transcription factors (as may occur in “enhanceosome” complexes [127]–[129]) could be involved. Computational algorithms more sophisticated than that used in the current study would be required to identify combinatorial transcription factor interactions. Such approaches in yeast have been described [130], [131], and if adapted to mammalian systems, these computational tools will likely help delineate the relative influence of synergistic transcription factor regulation.

While useful for predictive modeling, the construction of GRNs based on motif enrichment analysis has some limitations that must be considered when interpreting results. First, transcription factors of the same family (or even those more distantly related) can bind to homologous sites, leading to ambiguity in the modeling of the activity of specific transcription factors [60]. While some refinements of the model may be achieved by considering the absolute expression levels of individual transcription factors (i.e., a transcription factor with relatively abundant mRNA would be expected to contribute more to the network than one exhibiting low levels of transcription), these predictions must be validated experimentally. A second shortcoming is that regulatory elements outside the core promoter are not taken into account, nor is the chromatin arrangement of a given promoter. The latter omission might be reasonable, however, in light of genome wide studies of nucleosome arrangement, which suggest that the promoters of most genes reside in nucleosome-free regions (open chromatin configuration), and are permissive to transcription factor binding [63]. A third caveat is that motif enrichment analysis may be less effective when genes sharing the same cis-regulatory elements are not coexpressed, or when they are clustered incorrectly [90]. As discussed previously, we favored STEM clustering for analysis of our *in vitro* dataset, whereas the tissue-derived data (in which information about longitudinal expression was lacking) were clustered based upon overlap with the AMC data. Because we recognize that refinements in clustering of the Haddad *et al.* dataset of could influence the ultimate GRN model, our group is currently examining the utility of other clustering algorithms in this and other existing datasets. A fourth concern relates to the reliability of the motif enrichment prediction algorithms, since the number of predicted sites may exceed that of functional sites. In the present work, a high level of confidence could only be assigned to those transcription factors known to regulate target genes based on other reports (e.g., NF-κB). Although an effort was made to prune from our models those binding sites possessing a low degree of sequence similarity with canonical motifs (see **Figures 3** **and** **7**), this is limited to the single analytical method used. We recognize that while other means of motif enrichment analysis exist, this does not address the more fundamental issue that enrichment of binding sequences provides no information about whether or not a given motif is used. Finally, the construction of GRNs based on mRNA expression assumes that transcript levels reflect only the activity of transcription factors, and fails to consider important post-transcriptional regulatory mechanisms that likely influence mRNA abundance, such as microRNAs (miRNAs). The regulatory role of miRNAs is considerable, given that they are predicted to regulate more than one-third of all human genes, and could target as many as 200 transcripts each [132]. The prediction and modeling of post-transcriptional GRNs is an emerging area, and while the methods for modeling interactions between transcription factors, miRNAs, and genes are not as refined as those for transcriptional GRNs [133], the incorporation of such data would provide a more complete picture of the complex regulatory networks that exist among transcription factors, miRNAs, and genes. As we continue to extend our work, we intend to investigate the contributions of miRNAs to GRN models.

When extrapolating this approach to the experimental data obtained from the study of Haddad *et al.* [19], our primary goal was to infer regulatory relationships that might explain the observed transcriptional response to spontaneous term labor within the intrauterine environment. The choice of our *in vitro* model (sterile inflammation in primary amnion cells) was guided by the well-established association between inflammation in the fetal membranes and parturition [3]. While inflammation is associated with the process of parturition itself, it is generally accepted that pro-inflammatory biomolecules accumulate in the amniotic compartment near term, prior to the onset of active labor [134]. Currently, there is no consensus as to what the trigger(s) initiate this inflammatory cascade. In recent years, receptors responsive to pathogen-associated molecular patterns (PAMPs), in particular the TLRs, have received considerable attention as possible contributors to the labor-associated inflammatory response [135]. Multiple lines of evidence support this postulate: (1) microbial organisms, components of which activate TLRs, have been isolated from the amniotic cavity in approximately 19% of patients at term in labor with intact membranes [136]; (2) TLR expression increases within myometrium [85], [137] and fetal membranes [19], [138] with advancing gestation, and in term and preterm labor; (3) TLR polymorphisms have been associated with an increased risk of preterm labor [139], [140]; and (4) TLR ligands initiate labor in experimental animals [141], [142]. Our present work, however, suggests that TLR receptor activation, *per se*, could be dispensable for the majority of cases of term labor, given that our GRN model of labored fetal membranes (**Figure 8**) is indistinguishable from that of a sterile inflammatory transcriptional response [40]. Thus, it may be the case that our study provides potentially compelling evidence of a molecular distinction between normal, term parturition and infection-induced preterm labor. Signaling through the TLR4 receptor, which is responsive to Gram-negative lipopolysaccharide, may proceed through the MyD88-dependent pathway (like the IL-1 receptor), or via additional adapter proteins, such as TIR-domain-containing adapter-inducing interferon-β (TRIF) [38]. Stimulation of AMCs with IL-1β failed to evoke characteristic TRIF-regulated genes, such as *Ifnb1*, *Irg1*, *Ifit2*, and *Cxcl10* [81], [143]. Importantly, these genes were also absent from the datasets of Haddad *et al.* [19] and Bollopragada *et al.* [84], suggesting that signaling through TLR3 and/or TLR4 is not prominent in intrauterine tissues following term labor, at least among the cases selected for these studies which showed no signs of chorioamnionitis. While one cannot dismiss the potential for signaling through TLR2, TLR5, TLR7, or TLR9 (all MyD88-dependent [52], [144]) based on these results, we find that it is not necessary to invoke TLRs to explain transcriptional response following normal labor. We submit that sterile intrauterine inflammation, which may be elicited as a reaction to cellular debris [40], could contribute to the early inflammatory events leading to parturition. Indeed, ultrastructural analysis of the decidual component of full-thickness fetal membranes collected prior to term labor has revealed regions of cellular necrosis admixed with lipid-laden macrophages [145]; such areas could serve as foci for the production of pro-inflammatory biomolecules. In addition, sterile intrauterine inflammation may be amplified during active labor by oxidative stress, a result of frequent hypoxia-reoxygenation events caused by uterine contractions [75]–[78]. Fetal membrane hypoxia is supported by our finding that genes upregulated in response to labor have promoters that are enriched for HIF response elements (**Figures 7** **and** **8**).

The lack of an unambiguous TLR-mediated gene expression signature in the present analysis may be limited by the nature of the specimens queried. Certainly, biological variability among patients could conceal evidence of TLR-mediated gene expression, especially if such signaling is present only in a subset of cases. This could be assessed by examining additional clinical cases employing rigorous statistical analysis. The portions of the fetal membranes evaluated could also influence the observed transcriptional response. In the study of Haddad and colleagues [19], membranes were collected remote from the site of rupture, and specimens with histological evidence of chorioamnionitis were excluded. In a more recent analysis that examined global gene expression in different regions of term fetal membranes, 667 genes were found to be differentially expressed in the choriodecidua at the site of rupture relative to more distal regions [86]. While TRIF-dependent genes were not present among these differentially expressed genes, impact analysis revealed relative enrichment for the pathway of “pathogenic *Escherichia coli* infection” at the rupture site. Thus, signaling through TLRs could be regional in the fetal membranes, which is relevant given exposure of areas overlying the cervix (such as the membrane rupture site) to the vaginal microflora. Finally, it is also possible that marked leukocytic infiltration could lead to regulatory network models differing than that described here. Irrespective of the role of TLRs in term labor, however, there is some evidence that TLR-mediated signatures are present among the genes upregulated in the fetal membranes [2] and myometrium [74] following preterm labor, particularly when complicated by chorioamnionitis. As such, the GRNs governing preterm labor may differ from those involved in term labor. Deciphering the GRNs involved in preterm labor is an important extension of the current work, and ongoing studies by our group are directed at modeling global transcriptional control in this context.

Deciphering the complex physiological phenomenon of human parturition may be facilitated through incorporation of bioinformatic tools. The process presented here provides a facile means to infer transcriptional regulatory programs from high-dimensional datasets, and can be applied to the ever-expanding compendium of transcriptomic and proteomic information obtained from intrauterine tissues [146]–[150]. The GRN derived from the gene profiling study of Haddad and colleagues [19] suggests that term human labor resembles a sterile inflammatory response, and provides indirect evidence that components of the gene expression signature are likely to be governed by several inflammatory transcription factors, including NF-κB. The latter is relevant, given that in prior studies of fetal membranes in labor, direct evidence for NF-κB activation has been difficult to demonstrate conclusively [55], [151]–[153]. Importantly, this approach provides insight not only into the progression of labor, but also the upstream events that may govern labor's onset. In subsequent studies, GRN modeling may be helpful in elucidating among the proposed pathways leading to preterm birth [3], [154], [155].

At this point in time, it is important to emphasize the general approach, as one must appreciate that GRN construction is an iterative problem, for which refinements are mandatory. It remains that the development of highly veracious, global models of gene regulation is a formidable challenge in even relatively simple model systems, let alone in complex clinical tissue samples [91]. This is particularly so given that in tissues, there exist complex intercellular interactions that must be considered, along with limitations of specimen types [156], [157]. Thus, *in vivo* model development is complemented by data obtained using *in vitro* models, for which temporal expression data is available, and for which system complexity can be scaled-up in a rationale manner. Despite certain limitations in precision, GRN models based on gene expression profiling, clustering, and the prediction of *cis*-regulatory elements provide information that can be targeted in future studies, such as potential gene targets for chromatin immunoprecipitation (ChIP)-seq assays. Such models can then be extended to include data from other high-dimensional surveys, such as microRNA, ChIP-on-chip, and proteomics, providing more refined insight into global gene regulation.

## Supporting Information

Figure S1 **Verification of NF-κB-mediated gene activation using qRT-PCR.** Graphs showing the fold change of the mRNA expression levels of *Cxcl2*, *Il1b*, *Il6*, *Il8*, and *Ptgs2* in AMC cells pretreated with or without 30 µM of MG-132 prior to treatment with 10 ng/ml of IL-1β for 1 h (graphs A–E) and 8 h (graphs F–J). Control cells (veh) did not receive IL-1β treatment.
(TIF)

Figure S2 **Gene regulatory network of amnion mesenchymal cell dataset inferred from transcription factor binding motif results, detail of panel A in** **Figure 4** **.** Double circles represent binding motifs and ovals represent genes. Lines between motifs and genes represent inferred regulation based on Pscan motif analysis. The genes and respective connecting lines are colored based on the STEM profile groups depicted in Figure 1D (group A = red, group B = green, group C = blue, group D = black, group E = purple).
(TIF)

Figure S3 **Canonical pathways and biological functional analysis.** The top ten most significant canonical pathways and biological functions are listed for the fetal membrane data (black) and for the AMC data at 1 h (gray) and 8 h (white) post IL-1β treatment. The negative value of the log of the Benjamini Hochberg p-value is plotted for each function.
(TIF)

Table S1 **Target genes with microarray expression data.** This spreadsheet contains replicate-combined intensities for the full list of 190 unique, differentially expressed genes (see Materials and Methods, Microarray Analysis and Data Processing) from the microarray data of the amnion mesenchymal cells (AMCs). Column 1 indicates the HUGO gene symbol of the gene. Column 2 indicates the NCBI Entrez Gene ID. Column 3 provides the description of the gene, as based on the Affymetrix Human Genome GeneChip annotation file. Columns 4 through 6 indicate the average log_2_ intensity observed across three replicate experiments of vehicle control samples (Column 4), and AMCs treated with IL-1β for 1 h (Column 5) or 8 h (Column 6).
(XLS)

Table S2 **The temporal transcriptional response of amnion mesenchymal cells to cytokine challenge.** The significantly differentially expressed, unique genes (see Materials and Methods, Microarray Analysis and Data Processing) sampled at 0, 1, and 8 h following 10 ng/ml of IL-1β treatment were separated into 5 significantly represented profiles using STEM. The table is ordered by STEM profile (as illustrated in Figure 1C), then alphabetically by HUGO gene symbol. The 39 genes that did not fit into the top 5 profiles are listed as unassigned for their profile status. Column 1 indicates the HUGO gene symbol of the gene. Column 2 indicates the NCBI Entrez Gene ID. Column 3 contains the Affymetrix probeset(s) for the gene. Column 4 lists the profile for the gene while column 5 shows the coloring of the profile as shown in Figure 1C. Column 6 contains a description of the profile. Column 7 is the profile ID as given by the STEM software during analysis. Columns 8 through 10 indicates the STEM adjusted values of level of expression at 0 (column 8), 1 (column 9), and 8 h (column 10) post IL-1β treatment.
(XLS)

Table S3 **Transcription factor binding motif enrichment analysis for the AMC dataset of genes upregulated at 1 h or 8 h, as analyzed using Pscan.** Column 1 contains the names of JASPAR transcription factor matrices and column 2 contains the JASPAR matrix identification numbers. Column 3 contains the z-scores for each matrix as calculated with the z-test. The matrices are ranked by their p-value (column 4) that reflects the probability of having the same result by chance. Only matrices with p-values of less than 0.05 were considered to be potentially significant. Columns 5 through 8 contains the sample statistics for the input reference sequence mRNA accession numbers for the 169 genes (see Materials and Methods, Transcription Factor Binding Motif Enrichment Analysis) including average score in the input set (column 5) compared to the background mean (column 6) and standard deviation (column 7), along with input sample size (column 8).
(XLS)

Table S4 **Pscan results of transcription factor binding motif enrichment analysis using predicted non-NF-κB response genes from the AMC dataset.** Column 1 contains the names of JASPAR transcription factor matrices and column 2 contains the JASPAR matrix identification numbers. Column 3 contains the z-scores for each matrix as calculated with the z-test. The matrices are ranked by their p-value (column 4) that reflects the probability of having the same result by chance. Only matrices with p-values of less than 0.05 were considered to be potentially significant. Columns 5 through 8 contains the sample statistics for the input reference sequence mRNA accession numbers for the genes (see Materials and Methods, Transcription Factor Binding Motif Enrichment Analysis) including average score in the input set (column 5) compared to the background mean (column 6) and standard deviation (column 7), along with input sample size (column 8).
(XLS)

Table S5 **Nuclear receptor expression in amnion mesenchymal cells.** Column 1 indicates the HUGO gene symbol for nuclear receptors with probe sets represented on the Affymetrix GeneChip Human Genome U133A 2.0 Array used in this study. Column 2 provides the description of the gene. Column 3 indicates the expression level of the nuclear receptor based on the average log_2_ expression across all amnion mesenchymal cell (AMC) samples and replicates used in this study (column 5). Nuclear receptors with mean log_2_ expression values greater than the noise cut off value of 6 (see Materials and Methods, Microarray Analysis and Data Processing) were considered to be above noise (detectable mRNA expression), those with expression levels between 5.5 and 6 were considered to be borderline, and those with values below 5.5 were considered to be indistinguishable from background noise. Column 4 lists the evidence for the levels of expression of the nuclear receptors in AMCs. For peroxisome proliferator-activated receptor gamma and progesterone receptor, immunoblotting data (not shown) confirmed that the expression of these receptors was relatively deficient in AMCs when compared to cell lysates (differentiated 3T3-L1 mouse adipocytes and T47D human breast cancer cells, respectively) exhibiting positive expression.
(XLS)

Table S6 **Focus genes and top functions resulting from the network analysis, per network, for genes upregulated at 1 h post IL-1β treatment in AMCs.**
(PDF)

Table S7 **Focus genes and top functions resulting from the network analysis, per network, for genes upregulated at 8 h post IL-1β treatment in AMCs.**
(PDF)

Table S8 **Focus genes and top functions from the network analysis, per network, for genes differentially expressed in TIL versus TNL patients.**
(PDF)

## References

[1] Challis JR, Lockwood CJ, Myatt L, Norman JE, Strauss JF (2009). Inflammation and pregnancy.. Reprod Sci.

[2] Keelan JA, Blumenstein M, Helliwell RJ, Sato TA, Marvin KW (2003). Cytokines, prostaglandins and parturition-a
review.. Placenta.

[3] Romero R, Espinoza J, Goncalves LF, Kusanovic JP, Friel LA (2006). Inflammation in preterm and term labour and
delivery.. Semin Fetal Neonatal Med.

[4] Heaps BR, House M, Socrate S, Leppert P, Strauss JF, Petraglia F, Strauss JF, Gabbe SG, Weiss G (2007). Matrix biology and preterm birth.. Preterm birth: mechanisms, mediators, prediction, prevention and
interventions.

[5] Kennard EA, Zimmerman PD, Friedman CI, Kniss DA (1995). Interleukin-1 beta induces cyclooxygenase-2 in cultured human
decidual cells.. Am J Reprod Immunol.

[6] Kniss DA (1999). Cyclooxygenases in reproductive medicine and
biology.. J Soc Gynecol Investig.

[7] Perkins DJ, Kniss DA (1997). Tumor necrosis factor-alpha promotes sustained cyclooxygenase-2
expression: attenuation by dexamethasone and NSAIDs.. Prostaglandins.

[8] Lye SJ, Tsui P, Dong X, Mitchell J, Dorogin A, Petraglia F, Strauss JF, Gabbe SG, Weiss G (2007). Myometrial programming: a new concept underlying the regulation
of myometrial function during pregnancy.. Preterm birth: mechanisms, mediators, prediction, prevention and
interventions.

[9] Lappas M, Rice GE (2007). The role and regulation of the nuclear factor kappa B signalling
pathway in human labour.. Placenta.

[10] Lindstrom TM, Bennett PR (2005). The role of nuclear factor kappa B in human
labour.. Reproduction.

[11] Ackerman WE, Zhang XL, Rovin BH, Kniss DA (2005). Modulation of cytokine-induced cyclooxygenase 2 expression by
PPARG ligands through NFkappaB signal disruption in human WISH and amnion
cells.. Biol Reprod.

[12] Ackerman WE, Summerfield TL, Vandre DD, Robinson JM, Kniss DA (2008). Nuclear factor-kappa B regulates inducible prostaglandin E
synthase expression in human amnion mesenchymal cells.. Biol Reprod.

[13] Belt AR, Baldassare JJ, Molnar M, Romero R, Hertelendy F (1999). The nuclear transcription factor NF-kappaB mediates
interleukin-1beta-induced expression of cyclooxygenase-2 in human myometrial
cells.. Am J Obstet Gynecol.

[14] Kniss DA, Rovin B, Fertel RH, Zimmerman PD (2001). Blockade NF-kappaB activation prohibits TNF-alpha-induced
cyclooxygenase-2 gene expression in ED27 trophoblast-like
cells.. Placenta.

[15] Lappas M, Permezel M, Georgiou HM, Rice GE (2002). Nuclear factor kappa B regulation of proinflammatory cytokines in
human gestational tissues in vitro.. Biol Reprod.

[16] Jobin C, Sartor RB (2000). The I kappa B/NF-kappa B system: a key determinant of
mucosalinflammation and protection.. Am J Physiol Cell Physiol.

[17] Mendelson CR, Condon JC (2005). New insights into the molecular endocrinology of
parturition.. J Steroid Biochem Mol Biol.

[18] Whittle WL, Patel FA, Alfaidy N, Holloway AC, Fraser M (2001). Glucocorticoid regulation of human and ovine parturition: the
relationship between fetal hypothalamic-pituitary-adrenal axis activation
and intrauterine prostaglandin production.. Biol Reprod.

[19] Haddad R, Tromp G, Kuivaniemi H, Chaiworapongsa T, Kim YM (2006). Human spontaneous labor without histologic chorioamnionitis is
characterized by an acute inflammation gene expression
signature.. Am J Obstet Gynecol.

[20] Hassan SS, Romero R, Haddad R, Hendler I, Khalek N (2006). The transcriptome of the uterine cervix before and after
spontaneous term parturition.. Am J Obstet Gynecol.

[21] Iams JD (2003). Prediction and early detection of preterm labor.. Obstet Gynecol.

[22] Liggins GC (1988). The onset of labour: An overview.. The onset of labour: Cellular and integrative mechanisms 1-3.

[23] Norwitz ER, Robinson JN, Challis JR (1999). The control of labor.. N Engl J Med.

[24] Lang CT, Markham KB, Behrendt NJ, Suarez AA, Samuels P (2009). Placental dysferlin expression is reduced in severe
preeclampsia.. Placenta.

[25] R Development Core Team (2008). R: A Language and Environment for Statistical
Computing..

[26] Irizarry RA, Hobbs B, Collin F, Beazer-Barclay YD, Antonellis KJ (2003). Exploration, normalization, and summaries of high density
oligonucleotide array probe level data.. Biostatistics.

[27] Edgar R, Domrachev M, Lash AE (2002). Gene Expression Omnibus: NCBI gene expression and hybridization
array data repository.. Nucleic Acids Res.

[28] Smyth GK (2004). Linear models and empirical bayes methods for assessing
differential expression in microarray experiments.. Stat Appl Genet Mol Biol.

[29] Gordon A, Glazko G, Qiu X, Yakovlev A (2007). Control of the mean number of false discoveries, bonferroni and
stability of multiple testing.. Annals.

[30] Saeed AI, Sharov V, White J, Li J, Liang W (2003). TM4: a free, open-source system for microarray data management
and analysis.. Biotechniques.

[31] Ernst J, Bar-Joseph Z (2006). STEM: a tool for the analysis of short time series gene
expression data.. BMC Bioinformatics.

[32] Reimand J, Kull M, Peterson H, Hansen J, Vilo J (2007). g:Profiler-a web-based toolset for functional profiling of gene
lists from large-scale experiments.. Nucleic Acids Res.

[33] Zambelli F, Pesole G, Pavesi G (2009). Pscan: finding over-represented transcription factor binding site
motifs in sequences from co-regulated or co-expressed genes.. Nucleic Acids Res.

[34] Portales-Casamar E, Thongjuea S, Kwon AT, Arenillas D, Zhao X (2010). JASPAR 2010: the greatly expanded open-access database of
transcription factor binding profiles.. Nucleic Acids Res.

[35] Gansner ER, North SC (2000). An open graph visualization system and its applications to
software engineering.. Software: Practice and Experience.

[36] Livak KJ, Schmittgen TD (2001). Analysis of relative gene expression data using real-time
quantitative PCR and the 2(-Delta Delta C(T)) Method.. Methods.

[37] Carpenter S, O'Neill LA (2009). Recent insights into the structure of Toll-like receptors and
post-translational modifications of their associated signalling
proteins.. Biochem J.

[38] O'Neill LA (2008). The interleukin-1 receptor/Toll-like receptor superfamily: 10
years of progress.. Immunol Rev.

[39] Dinarello CA (2009). Interleukin-1beta and the autoinflammatory
diseases.. N Engl J Med.

[40] Rock KL, Latz E, Ontiveros F, Kono H (2010). The sterile inflammatory response.. Annu Rev Immunol.

[41] Perkins ND, Gilmore TD (2006). Good cop, bad cop: the different faces of
NF-kappaB.. Cell Death Differ.

[42] Smith C, Andreakos E, Crawley JB, Brennan FM, Feldmann M (2001). NF-kappaB-inducing kinase is dispensable for activation of
NF-kappaB in inflammatory settings but essential for lymphotoxin beta
receptor activation of NF-kappaB in primary human
fibroblasts.. J Immunol.

[43] Ochsenbein-Kolble N, Bilic G, Hall H, Huch R, Zimmermann R (2003). Inducing proliferation of human amnion epithelial and mesenchymal
cells for prospective engineering of membrane repair.. J Perinat Med.

[44] Holzinger D, Jorns C, Stertz S, Boisson-Dupuis S, Thimme R (2007). Induction of MxA gene expression by influenza A virus requires
type I or type III interferon signaling.. J Virol.

[45] Waddell SJ, Popper SJ, Rubins KH, Griffiths MJ, Brown PO (2010). Dissecting interferon-induced transcriptional programs in human
peripheral blood cells.. PLoS One.

[46] Haller O, Kochs G, Weber F (2006). The interferon response circuit: induction and suppression by
pathogenic viruses.. Virology.

[47] Koyama S, Ishii KJ, Coban C, Akira S (2008). Innate immune response to viral infection.. Cytokine.

[48] Wilkins C, Gale M (2010). Recognition of viruses by cytoplasmic sensors.. Curr Opin Immunol.

[49] Yao B, Rakhade SN, Li Q, Ahmed S, Krauss R (2004). Accuracy of cDNA microarray methods to detect small gene
expression changes induced by neuregulin on breast epithelial
cells.. BMC Bioinformatics.

[50] Sharif O, Bolshakov VN, Raines S, Newham P, Perkins ND (2007). Transcriptional profiling of the LPS induced NF-kappaB response
in macrophages.. BMC Immunol.

[51] Napolitani G, Rinaldi A, Bertoni F, Sallusto F, Lanzavecchia A (2005). Selected Toll-like receptor agonist combinations synergistically
trigger a T helper type 1-polarizing program in dendritic
cells.. Nat Immunol.

[52] Takeda K, Akira S (2004). TLR signaling pathways.. Semin Immunol.

[53] Akira S, Yamamoto M, Takeda K (2003). Role of adapters in Toll-like receptor
signalling.. Biochem Soc Trans.

[54] Chen LF, Greene WC (2004). Shaping the nuclear action of NF-kappaB.. Nat Rev Mol Cell Biol.

[55] Vora S, Abbas A, Kim CJ, Summerfield TL, Kusanovic JP (2010). Nuclear factor-kappa B localization and function within
intrauterine tissues from term and preterm labor and cultured fetal
membranes.. Reprod Biol Endocrinol.

[56] Ackerman WE, Rovin BH, Kniss DA (2004). Epidermal growth factor and interleukin-1beta utilize divergent
signaling pathways to synergistically upregulate cyclooxygenase-2 gene
expression in human amnion-derived WISH cells.. Biol Reprod.

[57] Kim SY, Kim Y (2006). Genome-wide prediction of transcriptional regulatory elements of
human promoters using gene expression and promoter analysis
data.. BMC Bioinformatics.

[58] Pahl HL (1999). Activators and target genes of Rel/NF-kappaB transcription
factors.. Oncogene.

[59] Ogawa S, Lozach J, Benner C, Pascual G, Tangirala RK (2005). Molecular determinants of crosstalk between nuclear receptors and
toll-like receptors.. Cell.

[60] Su G, Mao B, Wang J (2006). MACO: a gapped-alignment scoring tool for comparing transcription
factor binding sites.. In Silico Biol.

[61] Kaczynski J, Cook T, Urrutia R (2003). Sp1- and Kruppel-like transcription factors.. Genome Biol.

[62] Morris JF, Hromas R, Rauscher FJ, III (1994). Characterization of the DNA-binding properties of the myeloid
zinc finger protein MZF1: two independent DNA-binding domains recognize two
DNA consensus sequences with a common G-rich core.. Mol Cell Biol.

[63] Venters BJ, Pugh BF (2009). How eukaryotic genes are transcribed.. Crit Rev Biochem Mol Biol.

[64] Chabane N, Li X, Fahmi H (2009). HDAC4 contributes to IL-1-induced mPGES-1 expression in human
synovial fibroblasts through up-regulation of Egr-1 transcriptional
activity.. J Cell Biochem.

[65] Murakami M, Kudo I (2004). Recent advances in molecular biology and physiology of the
prostaglandin E2-biosynthetic pathway.. Prog Lipid Res.

[66] Wenke AK, Bosserhoff AK (2010). Roles of AP-2 transcription factors in the regulation of
cartilage and skeletal development.. FEBS J.

[67] Eckert D, Buhl S, Weber S, Jager R, Schorle H (2005). The AP-2 family of transcription factors.. Genome Biol.

[68] Hilger-Eversheim K, Moser M, Schorle H, Buettner R (2000). Regulatory roles of AP-2 transcription factors in vertebrate
development, apoptosis and cell-cycle control.. Gene.

[69] Pfisterer P, Ehlermann J, Hegen M, Schorle H (2002). A subtractive gene expression screen suggests a role of
transcription factor AP-2 alpha in control of proliferation and
differentiation.. J Biol Chem.

[70] MacKay C, Toth R, Rouse J (2009). Biochemical characterisation of the SWI/SNF family member
HLTF.. Biochem Biophys Res Commun.

[71] Unk I, Hajdu I, Fatyol K, Hurwitz J, Yoon JH (2008). Human HLTF functions as a ubiquitin ligase for proliferating cell
nuclear antigen polyubiquitination.. Proc Natl Acad Sci U S A.

[72] Johnsen O, Murphy P, Prydz H, Kolsto AB (1998). Interaction of the CNC-bZIP factor TCF11/LCR-F1/Nrf1 with MafG:
binding-site selection and regulation of transcription.. Nucleic Acids Res.

[73] Perera PM, Wypasek E, Madhavan S, Rath-Deschner B, Liu J (2010). Mechanical signals control SOX-9, VEGF, and c-Myc expression and
cell proliferation during inflammation via integrin-linked kinase, B-Raf,
and ERK1/2-dependent signaling in articular chondrocytes.. Arthritis Res Ther.

[74] Weiner CP, Mason CW, Dong Y, Buhimschi IA, Swaan PW (2010). Human effector/initiator gene sets that regulate myometrial
contractility during term and preterm labor.. Am J Obstet Gynecol.

[75] Fleischer A, Anyaegbunam AA, Schulman H, Farmakides G, Randolph G (1987). Uterine and umbilical artery velocimetry during normal
labor.. Am J Obstet Gynecol.

[76] Brar HS, Platt LD, DeVore GR, Horenstein J, Medearis AL (1988). Qualitative assessment of maternal uterine and fetal umbilical
artery blood flow and resistance in laboring patients by Doppler
velocimetry.. Am J Obstet Gynecol.

[77] Cindrova-Davies T, Yung HW, Johns J, Spasic-Boskovic O, Korolchuk S (2007). Oxidative stress, gene expression, and protein changes induced in
the human placenta during labor.. Am J Pathol.

[78] Cindrova-Davies T, Spasic-Boskovic O, Jauniaux E, Charnock-Jones DS, Burton GJ (2007). Nuclear factor-kappa B, p38, and stress-activated protein kinase
mitogen-activated protein kinase signaling pathways regulate proinflammatory
cytokines and apoptosis in human placental explants in response to oxidative
stress: effects of antioxidant vitamins.. Am J Pathol.

[79] Hung TH, Charnock-Jones DS, Skepper JN, Burton GJ (2004). Secretion of tumor necrosis factor-alpha from human placental
tissues induced by hypoxia-reoxygenation causes endothelial cell activation
in vitro: a potential mediator of the inflammatory response in
preeclampsia.. Am J Pathol.

[80] Brazma A, Jonassen I, Vilo J, Ukkonen E (1998). Predicting gene regulatory elements in silico on a genomic
scale.. Genome Res.

[81] Ramsey SA, Klemm SL, Zak DE, Kennedy KA, Thorsson V (2008). Uncovering a macrophage transcriptional program by integrating
evidence from motif scanning and expression dynamics.. PLoS Comput Biol.

[82] Coppe A, Ferrari F, Bisognin A, Danieli GA, Ferrari S (2009). Motif discovery in promoters of genes co-localized and
co-expressed during myeloid cells differentiation.. Nucleic Acids Res.

[83] Hassan SS, Romero R, Tarca AL, Nhan-Chang CL, Vaisbuch E (2009). The transcriptome of cervical ripening in human pregnancy before
the onset of labor at term: identification of novel molecular functions
involved in this process.. J Matern Fetal Neonatal Med.

[84] Bollapragada S, Youssef R, Jordan F, Greer I, Norman J (2009). Term labor is associated with a core inflammatory response in
human fetal membranes, myometrium, and cervix.. Am J Obstet Gynecol.

[85] Mittal P, Romero R, Tarca AL, Gonzalez J, Draghici S (2010). Characterization of the myometrial transcriptome and biological
pathways of spontaneous human labor at term.. J Perinat Med.

[86] Nhan-Chang CL, Romero R, Tarca AL, Mittal P, Kusanovic JP (2010). Characterization of the transcriptome of chorioamniotic membranes
at the site of rupture in spontaneous labor at term.. Am J Obstet Gynecol.

[87] Khanjani S, Kandola MK, Lindstrom TM, Sooranna SR, Melchionda M (2010). NF-kappaB regulates a cassette of immune/inflammatory genes in
human pregnant myometrium at term.. J Cell Mol Med.

[88] Wognum S, Lagoa CE, Nagatomi J, Sacks MS, Vodovotz Y (2009). An exploratory pathways analysis of temporal changes induced by
spinal cord injury in the rat bladder wall: insights on remodeling and
inflammation.. PLoS One.

[89] Elbers CC, van Eijk KR, Franke L, Mulder F, van der Schouw YT (2009). Using genome-wide pathway analysis to unravel the etiology of
complex diseases.. Genet Epidemiol.

[90] Lee NH (2005). Genomic approaches for reconstructing gene
networks.. Pharmacogenomics.

[91] Goutsias J, Lee NH (2007). Computational and experimental approaches for modeling gene
regulatory networks.. Curr Pharm Des.

[92] Schlitt T, Brazma A (2007). Current approaches to gene regulatory network
modelling.. BMC Bioinformatics.

[93] Lee WP, Tzou WS (2009). Computational methods for discovering gene networks from
expression data.. Brief Bioinform.

[94] Haverty PM, Hansen U, Weng Z (2004). Computational inference of transcriptional regulatory networks
from expression profiling and transcription factor binding site
identification.. Nucleic Acids Res.

[95] Chua G, Robinson MD, Morris Q, Hughes TR (2004). Transcriptional networks: reverse-engineering gene regulation on
a global scale.. Curr Opin Microbiol.

[96] Zhang MQ (2007). Computational analyses of eukaryotic promoters.. BMC Bioinformatics.

[97] He F, Balling R, Zeng AP (2009). Reverse engineering and verification of gene networks:
principles, assumptions, and limitations of present methods and future
perspectives.. J Biotechnol.

[98] Li H, Xuan J, Wang Y, Zhan M (2008). Inferring regulatory networks.. Front Biosci.

[99] Segal E, Shapira M, Regev A, Pe'er D, Botstein D (2003). Module networks: identifying regulatory modules and their
condition-specific regulators from gene expression data.. Nat Genet.

[100] Yan X, Mehan MR, Huang Y, Waterman MS, Yu PS (2007). A graph-based approach to systematically reconstruct human
transcriptional regulatory modules.. Bioinformatics.

[101] Alter O, Brown PO, Botstein D (2000). Singular value decomposition for genome-wide expression data
processing and modeling.. Proc Natl Acad Sci U S A.

[102] Lee SI, Batzoglou S (2003). Application of independent component analysis to
microarrays.. Genome Biol.

[103] Frigyesi A, Veerla S, Lindgren D, Hoglund M (2006). Independent component analysis reveals new and biologically
significant structures in micro array data.. BMC Bioinformatics.

[104] Omberg L, Golub GH, Alter O (2007). A tensor higher-order singular value decomposition for
integrative analysis of DNA microarray data from different
studies.. Proc Natl Acad Sci U S A 20;.

[105] Li H, Sun Y, Zhan M (2007). The discovery of transcriptional modules by a two-stage matrix
decomposition approach.. Bioinformatics.

[106] Bar-Joseph Z, Gerber GK, Lee TI, Rinaldi NJ, Yoo JY (2003). Computational discovery of gene modules and regulatory
networks.. Nat Biotechnol.

[107] Zhu Y, Li H, Miller DJ, Wang Z, Xuan J (2008). caBIG VISDA: modeling, visualization, and discovery for cluster
analysis of genomic data.. BMC Bioinformatics.

[108] Elnitski L, Jin VX, Farnham PJ, Jones SJ (2006). Locating mammalian transcription factor binding sites: a survey
of computational and experimental techniques.. Genome Res.

[109] van NE (2007). Finding regulatory elements and regulatory motifs: a general
probabilistic framework.. BMC Bioinformatics.

[110] Brown CT (2008). Computational approaches to finding and analyzing cis-regulatory
elements.. Methods Cell Biol.

[111] Das MK, Dai HK (2007). A survey of DNA motif finding algorithms.. BMC Bioinformatics.

[112] Zheng J, Wu J, Sun Z (2003). An approach to identify over-represented cis-elements in related
sequences.. Nucleic Acids Res.

[113] Sinha S, Tompa M (2003). YMF: A program for discovery of novel transcription factor
binding sites by statistical overrepresentation.. Nucleic Acids Res.

[114] Frith MC, Fu Y, Yu L, Chen JF, Hansen U (2004). Detection of functional DNA motifs via statistical
over-representation.. Nucleic Acids Res.

[115] Ho Sui SJ, Mortimer JR, Arenillas DJ, Brumm J, Walsh CJ (2005). oPOSSUM: identification of over-represented transcription factor
binding sites in co-expressed genes.. Nucleic Acids Res.

[116] Marinescu VD, Kohane IS, Riva A (2005). MAPPER: a search engine for the computational identification of
putative transcription factor binding sites in multiple
genomes.. BMC Bioinformatics.

[117] Hestand MS, van GM, Villerius MP, van Ommen GJ, den Dunnen JT (2008). CORE_TF: a user-friendly interface to identify evolutionary
conserved transcription factor binding sites in sets of co-regulated
genes.. BMC Bioinformatics.

[118] Tonon L, Touzet H, Varre JS (2010). TFM-Explorer: mining cis-regulatory regions in genomes. Nucleic
Acids Res 38 Suppl:W286-92.. Epub 2010 Jun.

[119] Chang LW, Fontaine BR, Stormo GD, Nagarajan R (2007). PAP: a comprehensive workbench for mammalian transcriptional
regulatory sequence analysis.. Nucleic Acids Res.

[120] Roider HG, Manke T, O'Keeffe S, Vingron M, Haas SA (2009). PASTAA: identifying transcription factors associated with sets of
co-regulated genes.. Bioinformatics.

[121] Cora D, Herrmann C, Dieterich C, Di CF, Provero P (2005). Ab initio identification of putative human transcription factor
binding sites by comparative genomics.. BMC Bioinformatics.

[122] Khatri P, Desai V, Tarca AL, Sellamuthu S, Wildman DE (2006). New Onto-Tools: Promoter-Express, nsSNPCounter and
Onto-Translate.. Nucleic Acids Res.

[123] Posch S, Grau J, Gohr A, Ben-Gal I, Kel AE (2007). Recognition of cis-regulatory elements with
vombat.. J Bioinform Comput Biol.

[124] Lee YW, Kuhn H, Hennig B, Neish AS, Toborek M (2001). IL-4-induced oxidative stress upregulates VCAM-1 gene expression
in human endothelial cells.. J Mol Cell Cardiol.

[125] Giorgetti L, Siggers T, Tiana G, Caprara G, Notarbartolo S (2010). Noncooperative interactions between transcription factors and
clustered DNA binding sites enable graded transcriptional responses to
environmental inputs.. Mol Cell.

[126] Tay S, Hughey JJ, Lee TK, Lipniacki T, Quake SR (2010). Single-cell NF-kappaB dynamics reveal digital activation and
analogue information processing.. Nature.

[127] Carey M (1998). The enhanceosome and transcriptional synergy.. Cell.

[128] Tsai EY, Falvo JV, Tsytsykova AV, Barczak AK, Reimold AM (2000). A lipopolysaccharide-specific enhancer complex involving Ets,
Elk-1, Sp1, and CREB binding protein and p300 is recruited to the tumor
necrosis factor alpha promoter in vivo.. Mol Cell Biol.

[129] Merika M, Thanos D (2001). Enhanceosomes.. Curr Opin Genet Dev.

[130] Bussemaker HJ, Li H, Siggia ED (2001). Regulatory element detection using correlation with
expression.. Nat Genet.

[131] Pilpel Y, Sudarsanam P, Church GM (2001). Identifying regulatory networks by combinatorial analysis of
promoter elements.. Nat Genet.

[132] Krek A, Grun D, Poy MN, Wolf R, Rosenberg L (2005). Combinatorial microRNA target predictions.. Nat Genet.

[133] Gunaratne PH, Creighton CJ, Watson M, Tennakoon JB (2010). Large-scale integration of MicroRNA and gene expression data for
identification of enriched microRNA-mRNA associations in biological
systems.. Methods Mol Biol.

[134] Romero R, Munoz H, Gomez R, Parra M, Polanco M (1996). Increase in prostaglandin bioavailability precedes the onset of
human parturition.. Prostaglandins Leukot Essent Fatty Acids.

[135] Young SL (2009). The “toll” of labor.. Reprod Sci.

[136] Romero R, Nores J, Mazor M, Sepulveda W, Oyarzun E (1993). Microbial invasion of the amniotic cavity during term labor.
Prevalence and clinical significance.. J Reprod Med.

[137] Youssef RE, Ledingham MA, Bollapragada SS, O'Gorman N, Jordan F (2009). The role of toll-like receptors (TLR-2 and -4) and triggering
receptor expressed on myeloid cells 1 (TREM-1) in human term and preterm
labor.. Reprod Sci.

[138] Kim YM, Romero R, Chaiworapongsa T, Kim GJ, Kim MR (2004). Toll-like receptor-2 and -4 in the chorioamniotic membranes in
spontaneous labor at term and in preterm parturition that are associated
with chorioamnionitis.. Am J Obstet Gynecol.

[139] Rey G, Skowronek F, Alciaturi J, Alonso J, Bertoni B (2008). Toll receptor 4 Asp299Gly polymorphism and its association with
preterm birth and premature rupture of membranes in a South American
population.. Mol Hum Reprod.

[140] Krediet TG, Wiertsema SP, Vossers MJ, Hoeks SB, Fleer A (2007). Toll-like receptor 2 polymorphism is associated with preterm
birth.. Pediatr Res.

[141] Haddad R, Gould BR, Romero R, Tromp G, Farookhi R (2006). Uterine transcriptomes of bacteria-induced and
ovariectomy-induced preterm labor in mice are characterized by differential
expression of arachidonate metabolism genes.. Am J Obstet Gynecol.

[142] Ilievski V, Lu SJ, Hirsch E (2007). Activation of toll-like receptors 2 or 3 and preterm delivery in
the mouse.. Reprod Sci.

[143] Hirotani T, Yamamoto M, Kumagai Y, Uematsu S, Kawase I (2005). Regulation of lipopolysaccharide-inducible genes by MyD88 and
Toll/IL-1 domain containing adaptor inducing IFN-beta.. Biochem Biophys Res Commun.

[144] Kawai T, Akira S (2005). Toll-like receptor downstream signaling.. Arthritis Res Ther.

[145] Ackerman WE, Robinson JM, Kniss DA (2007). Association of PAT proteins with lipid storage droplets in term
fetal membranes.. Placenta.

[146] Romero R, Espinoza J, Gotsch F, Kusanovic JP, Friel LA (2006). The use of high-dimensional biology (genomics, transcriptomics,
proteomics, and metabolomics) to understand the preterm parturition
syndrome.. BJOG 113 Suppl.

[147] Robinson JM, Ackerman WE, Kniss DA, Takizawa T, Vandre DD (2008). Proteomics of the human placenta: promises and
realities.. Placenta.

[148] Robinson JM, Vandre DD, Ackerman WE (2009). Placental proteomics: a shortcut to biological
insight.. Placenta.

[149] Zhang C, Han L, Zhang A, Yang W, Zhou X (2010). Global changes of mRNA expression reveals an increased activity
of the interferon-induced signal transducer and activator of transcription
(STAT) pathway by repression of miR-221/222 in glioblastoma U251
cells.. Int J Oncol.

[150] Shankar R, Johnson MP, Williamson NA, Cullinane F, Purcell AW (2010). Molecular markers of preterm labor in the
choriodecidua.. Reprod Sci.

[151] Yan X, Sun M, Gibb W (2002). Localization of nuclear factor-kappa B (NF kappa B) and
inhibitory factor-kappa B (I kappa B) in human fetal membranes and decidua
at term and preterm delivery.. Placenta.

[152] Mitchell CM, Johnson RF, Giles WB, Zakar T (2008). Prostaglandin H synthase-2 gene regulation in the amnion at
labour: histone acetylation and nuclear factor kappa B binding to the
promoter in vivo.. Mol Hum Reprod.

[153] Lappas M, Rice GE (2009). Transcriptional regulation of the processes of human labour and
delivery..

[154] Lockwood CJ, Kuczynski E (2001). Risk stratification and pathological mechanisms in preterm
delivery.. Paediatr Perinat Epidemiol.

[155] Goldenberg RL, Iams JD, Mercer BM, Meis PJ, Moawad AH (1998). The preterm prediction study: the value of new vs standard risk
factors in predicting early and all spontaneous preterm births. NICHD MFMU
Network.. Am J Public Health.

[156] Dobrin R, Zhu J, Molony C, Argman C, Parrish ML (2009). Multi-tissue coexpression networks reveal unexpected subnetworks
associated with disease.. Genome Biol.

[157] Kirouac DC, Ito C, Csaszar E, Roch A, Yu M (2010). Dynamic interaction networks in a hierarchically organized
tissue.. Mol Syst Biol.

