# Supplementary figures and images for: Inflammatory Gene Regulatory Networks in Amnion Cells Following Cytokine Stimulation: Translational Systems Approach to Modeling Human Parturition
Source: PLoS One. 2011 Jun 2;6(6):e20560. doi: 10.1371/journal.pone.0020560 (PMC3107214; doi:10.1371/journal.pone.0020560)

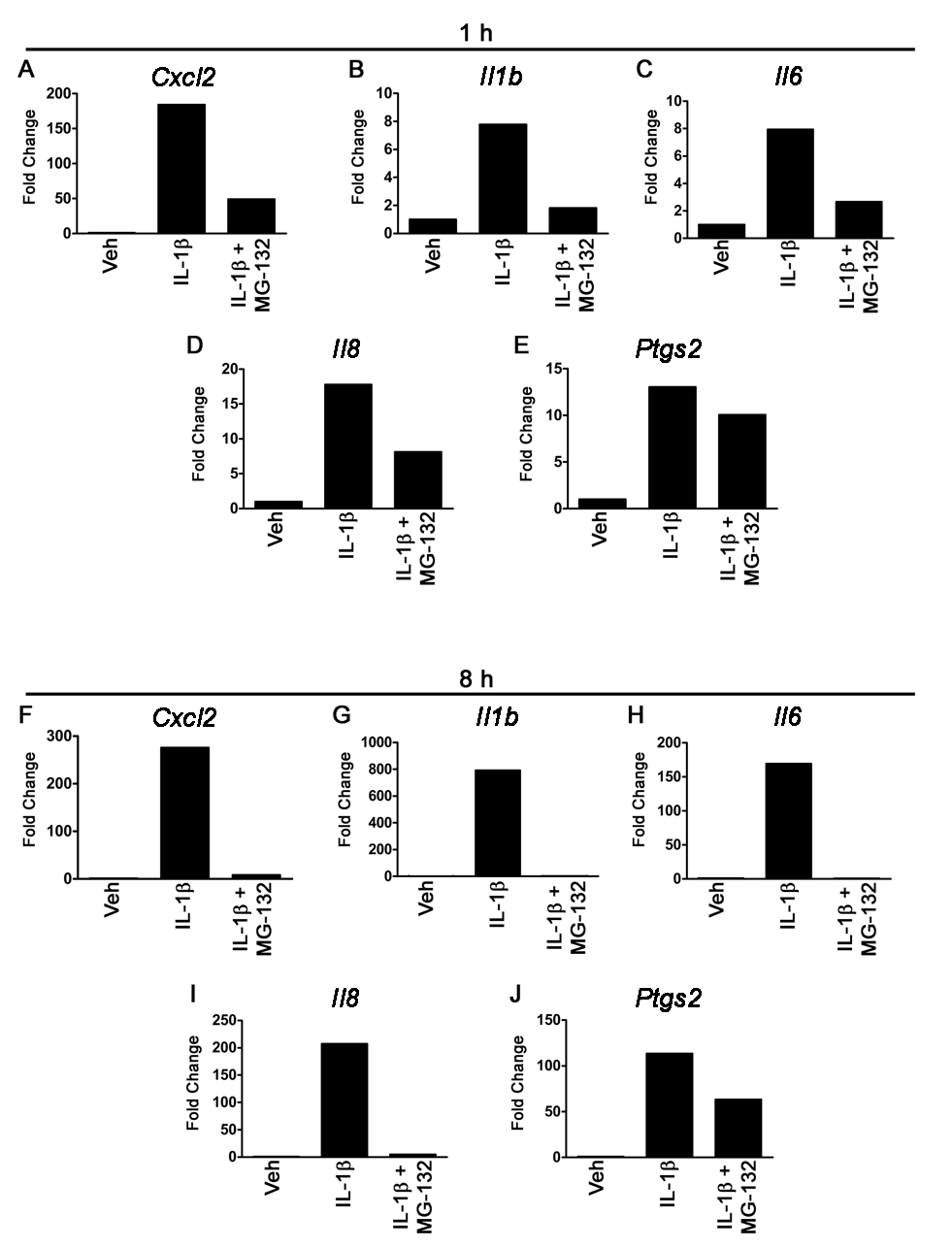

Supplement: Figure S1 — Verification of NF-κB-mediated gene activation using qRT-PCR. Graphs showing the fold change of the mRNA expression levels of Cxcl2, Il1b, Il6, Il8, and Ptgs2 in AMC cells pretreated with or without 30 µM of MG-132 prior to treatment with 10 ng/ml of IL-1β for 1 h (graphs A–E) and 8 h (graphs F–J). Control cells (veh) did not receive IL-1β treatment. (TIF) [file pone.0020560.s001.tif]

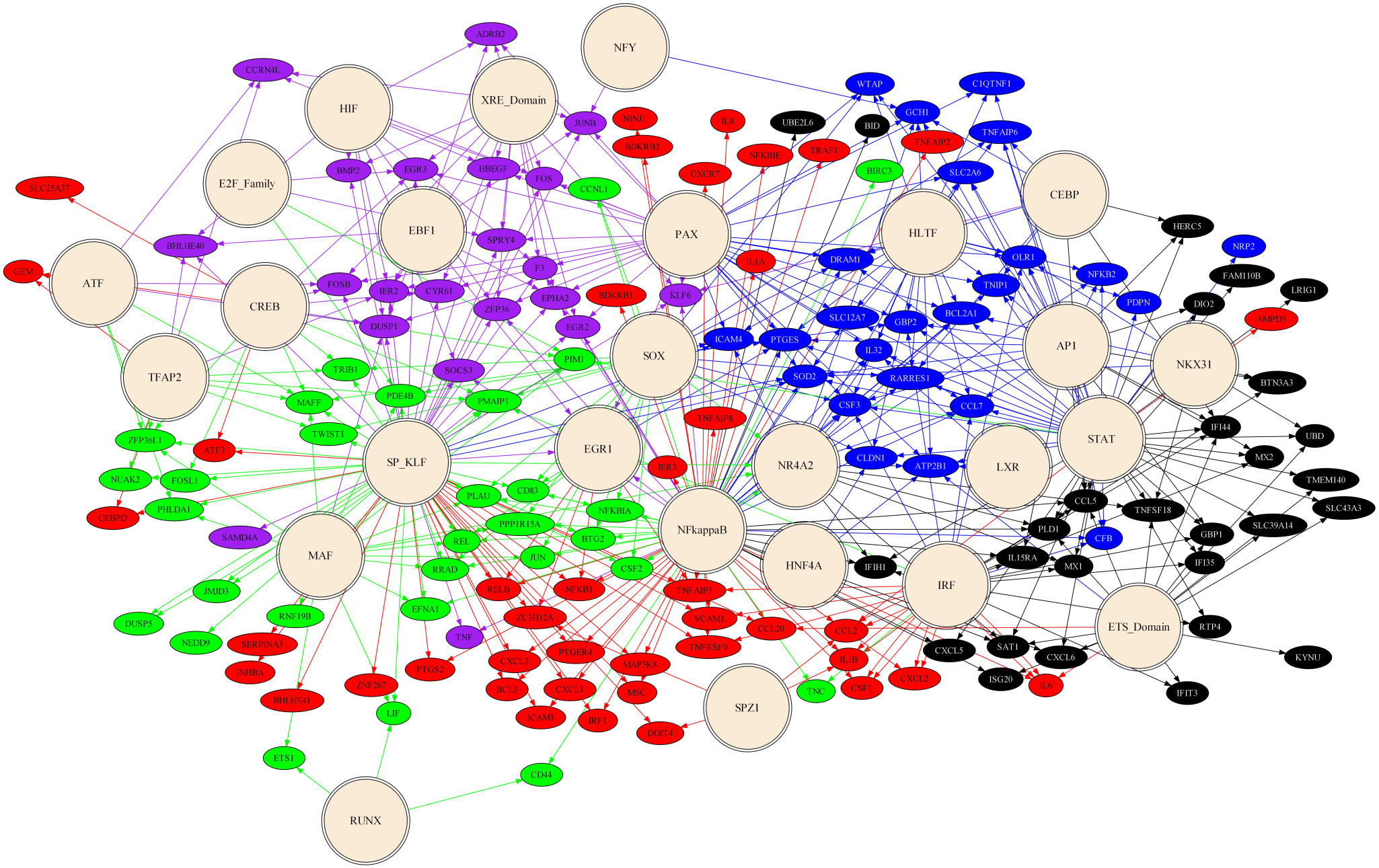

Supplement: Figure S2 — Gene regulatory network of amnion mesenchymal cell dataset inferred from transcription factor binding motif results, detail of panel A in Figure 4 . Double circles represent binding motifs and ovals represent genes. Lines between motifs and genes represent inferred regulation based on Pscan motif analysis. The genes and respective connecting lines are colored based on the STEM profile groups depicted in Figure 1D (group A = red, group B = green, group C = blue, group D = black, group E = purple). (TIF) [file pone.0020560.s002.tif]

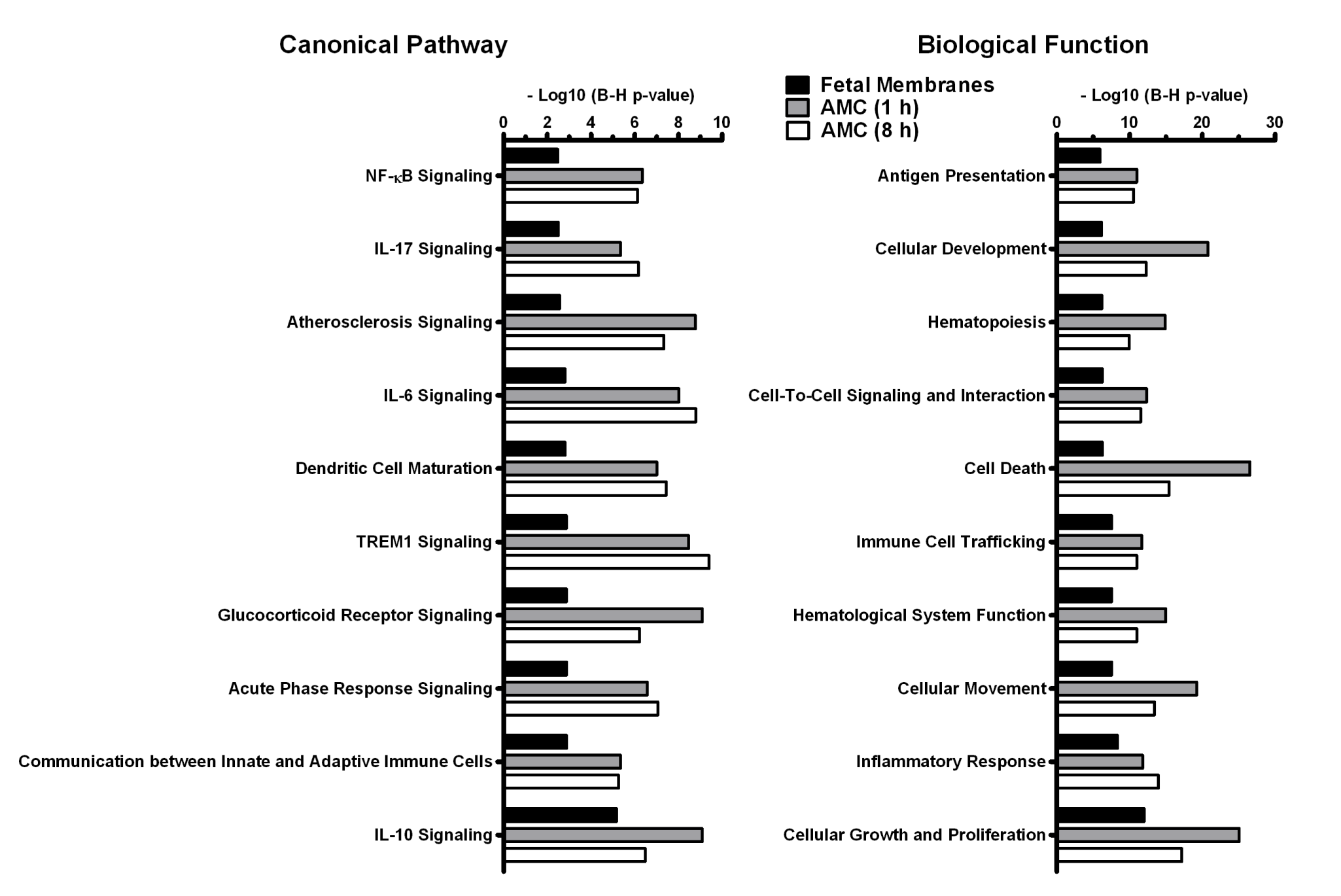

Supplement: Figure S3 — Canonical pathways and biological functional analysis. The top ten most significant canonical pathways and biological functions are listed for the fetal membrane data (black) and for the AMC data at 1 h (gray) and 8 h (white) post IL-1β treatment. The negative value of the log of the Benjamini Hochberg p-value is plotted for each function. (TIF) [file pone.0020560.s003.tif]
