# Supplementary material for: Inflammatory Gene Regulatory Networks in Amnion Cells Following Cytokine Stimulation: Translational Systems Approach to Modeling Human Parturition
Source: PLoS One. 2011 Jun 2;6(6):e20560. doi: 10.1371/journal.pone.0020560 (PMC3107214; doi:10.1371/journal.pone.0020560)
Supplement: Table S6 — Focus genes and top functions resulting from the network analysis, per network, for genes upregulated at 1 h post IL-1β treatment in AMCs. (PDF) [file pone.0020560.s009.pdf]

**Table S6.** Focus genes and top functions resulting from the network analysis, per network, for genes upregulated at 1 h post IL-1β treatment in AMCs.

| No. | Molecules in Network | Score | Focus Genes | Top Functions |
|---|---|---|---|---|
| 1 | CHEMOKINE,CXCL1,CXCL2,CXCL3,CXCR7,DUSP2,DUSP5,ETS,GCH1,I kappa b kinase,Icam,ICAM4 (includes EG:3386),IER2,Ifn,Ikk (family), IL1/IL6/TNF,IRF,MAFF, MSC,NFkB (family),NfkB-RelA,NFKBIE,NKX3-1,NUAK2,OLR1,PLK3, Pro-inflammatory Cytokine,PTX3,SDC4,Tlr,TNF,TNFAIP2,TNFAIP8,Ube3, ZNF267 | 44 | 22 | Neurological Disease, Cellular Growth and Proliferation, Nervous System Development and Function |
| 2 | ATF3,Caspase 3/7, Cbp/p300, CCNL1,CEBPD,Cyclooxygenase, DUSP1, DUSP6, EGR1, Eotaxin,ERK1/2,ETS1,FOSB,FOSL1,GC-GCR dimer,Growth hormone,IL6, JAK,JINK1/2, JUNB,LIF,N-cor,NR4A2,PDGF BB, PHLDA1, PTGER4,Rar,Rxr, Smad2/3,SOCS,SOCS3, STAT,STAT5a/b,Thyroid hormone receptor,TNC | 32 | 17 | Organ Morphology, Reproductive System Development and Function, Cellular Growth and Proliferation |
| 3 | BCL3,BCL2A1,BIRC3,CCL20,EFNA1,F3,Fibrinogen,Glucocorticoid-GCR,IER3,Ikb, IKK (complex),IL23,IL1A,IRF1,LYMPHOTOXIN-ALPHA1-BETA2,MHC CLASS I (family), NFKB1,NFkB (complex),NfkB1-RelA,NFKBIA, PMAIP1,PPARÎ±-RXRÎ±,REL, REL/RELA/RELB,RELB,SAA@, SPHINGOMYELINASE,STAT1/3/5 dimer, Stat3-Stat3, SWI-SNF,Tnf receptor, TNFAIP3,TRAF,TRAF1,ZFP36 | 31 | 17 | Gene Expression, Cell Death, Inflammatory Response |
| 4 | ALP,Ap1,BHLHE40,BHLHE41,BMP2,CD83,Collagen type I,Cpla2,CSF2, Estrogen Receptor,GADD45B,HLA-DR,Hsp27,Hsp70,IFN Beta,Ifn gamma,IgG, IL1,IL12 (complex), INHBA,Interferon alpha,KDM6B,LDL,MHC Class II,Nos,P38 MAPK,PI3K,PIM1, PPP1R15A (includes EG:23645),PTGS2,Sapk,Smad,Tgf beta,TRIB1,TWIST1 | 22 | 13 | Cell Morphology, Tissue Morphology, Cell-To-Cell Signaling and Interaction |
| 5 | ALOX5,ANKRD1,BUB1,CCR2,CD55,CDKN2C,CKS1B,CXCL3,DDIT4,EGR3,ELK3,EPHA2,FOSL1,GDF15,HBEGF,hydrogen peroxide,KIT,KLF9,lipoxin A4, MUC5AC,NR4A3, PLA2G4A,PTGER4,RNF19B,SERPINH1,Sod,TCF3,TFDP1,TGFB1,TIAL1,Timp,TXNIP, VCAM1,ZC3H12A,ZFP36 | 19 | 12 | Cellular Growth and Proliferation, Cell Death, Cardiovascular System Development and Function |
| 6 | BDKRB1,C8,C1q,Calcineurin A,CCL2,CD44,Collagen(s),CSF1,CYR61,Elastase,Erm, Fibrin,Focal adhesion kinase,Gpcr,HBEGF,ICAM1,IL1B,JUN/JUNB/JUND,KLF6,Laminin, Mmp,Neurotrophin,Nfat (family),NGF,P110,p70 S6k,Pdgf,PLAU, RCAN1,Secretase gamma,Shc,Sod,Sphk,VCAM1,Vegf | 17 | 12 | Cardiovascular System Development and Function, Cell-To-Cell Signaling and Interaction, Tissue Development |
| 7 | ADRB2,Akt,BTG2,Calmodulin,Calpain,CaMKII,CD3,Creb,EGR2,ERK,Fgf,FOS,hCG,Integrin,JUN,MAP2K1/2,MAP3K8,Mapk,Mek,PDE4B,Pias,Pka,Pkc(s),PLA2,PLC,PP2A,Rac,Rap1,Ras, Ras homolog,RGS2,SPRY2,SPRY4,TCR,TMSB4 | 16 | 10 | Cellular Development, Cell Signaling, Nucleic Acid Metabolism |

# Only the top 7 scoring networks are depicted.
