# Supplementary material for: Inflammatory Gene Regulatory Networks in Amnion Cells Following Cytokine Stimulation: Translational Systems Approach to Modeling Human Parturition
Source: PLoS One. 2011 Jun 2;6(6):e20560. doi: 10.1371/journal.pone.0020560 (PMC3107214; doi:10.1371/journal.pone.0020560)
Supplement: Table S7 — Focus genes and top functions resulting from the network analysis, per network, for genes upregulated at 8 h post IL-1β treatment in AMCs. (PDF) [file pone.0020560.s010.pdf]

Table S7. Focus genes and top functions resulting from the network analysis, per network, for genes upregulated at 8 h post IL-1β treatment in AMCs.

No.	Molecules in Network	Score	Focus Genes	Top Functions
1	CCL7,CHEMOKINE,CXCL1,CXCL2,CXCL3,CXCL5,CXCL6,ETS,GCH1,GFPT2,IFIH1,Ifn, IFN TYPE 1,IL1/IL6/TNF,IL7R,IRF,LYMPHOTOXIN-ALPHA1-BETA2,NFkB (complex),NFkB (family),NfkB-RelA,NUAK2,OLR1,PPARÎ±-RXRÎ±,Pro-inflammatory Cytokine, REL/RELA/RELB,SLC12A7,SLC2A6,Tlr,TNFAIP2,TNFAIP6,TNFAIP8, TNFRSF9,TNFSF18,TNIP1,WTAP	41	21	Cell-To-Cell Signaling and Interaction, Hematological System Development and Function, Antigen Presentation
2	BIRC3,BTN3A3,C2,CCL20,CSF3,Cyclooxygenase,Elastase,Eotaxin,ERK1/2,GBP1 (includes EG:2633),Ikb,IKK (complex),Ikk (family),IL1,IL23,IL32,IL17R,IL1A,IL1B, JINK1/2,Mmp,MMP10,MMP12,NfkB1-RelA,PTGER4,PTGES,RIPK2,SAA@, SERPINA3,TMSB4, Tnf receptor,TNFAIP3,TNFRSF11B,TRAF,TRAF1	32	18	Inflammatory Response, Organismal Functions, Connective Tissue Disorders
3	26s Proteasome,BCL3,CCL5,CD83,Collagen type I,Cpla2,CSF1,CSF2,HLA-DR,I kappa b kinase,IER3,IFI,IFI35,IFI44,IFN Beta,Ifn gamma,IL12 (complex),Interferon alpha,IRF1, JAK,LDL,MHC Class II,MX1,MX2,NAMPT,NFKB2,NFKBIE,P110,PI3K,REL,RELB, SPHINGOMYELINASE,STAT,STAT5a/b,SWI-SNF	28	16	Cellular Development, Antigen Presentation, Hematological System Development and Function
4	AMPD3,C1QTNF1,CLDN1,CXCL3,DDIT4,DRAM1,ENPP1,FAM110B,GBP1 (includes EG:2633),HAS1,Histone h4,hydrogen peroxide,IL1B,IL1F9,ITPA,KIAA0247,MMP2, NAB1,NLRC4,NR3C1,NRP2,OLR1,PDLIM4,PIM2 (includes EG:11040),SEPP1, SERPINH1,SLC39A14,SRGN,TGFB1,THBS2,Timp,TP53,TYMP,UGDH,VEGFA	27	16	Cellular Development, Cell Death, Cardiovascular System Development and Function
5	ALP,Ap1,ATF3,BCL2A1,BDKRB1,BDKRB2,Calpain,Cbp/p300,CCL2,CEBPD,Collagen(s),Cyclin E,FGF2,Fibrinogen,Focal adhesion kinase,GEM,IL6,INHBA,Laminin,LIF, MAP2K1/2,Mek,N-cor,p70 S6k,Pdgf,PDGF BB,PTGS2,Rar,Ras,Rxr,Smad,Smad2/3, Sphk,Tgf beta,TNC	22	13	Organ Morphology, Reproductive System Development and Function, Cellular Growth and Proliferation
6	ARFGAP3,CD276,CD1E,CHI3L2,CHST2,CLEC4E,CXCR7,dihydrotestosterone,ERBB2, ETV1,FCGR1B,IFNG,IL13,IL17B,IL17C,L-tryptophan,LRIG1,MSC,NFAM1,NINJ1,NKX3-1, RARRES1,RTP4,SCUBE1,SEPP1,SLC22A16,SLC25A37,SLC43A3,SLC7A7,SQLE, TCF3,TDO2,TNF,ZC3H12A,ZNF267	22	13	Cell-To-Cell Signaling and Interaction, Cellular Growth and Proliferation, Small Molecule Biochemistry
7	Akt,BID,Caspase,CD3,Creb,Cyclin A,DIO2,Estrogen Receptor,hCG,Histone h3,Hsp27, Hsp70,Icam,ICAM1,ICAM4 (includes EG:3386),ID1,IL8,JUN,MAFF,MAP3K8,Nfat (family),NFKBIA,NKX3-1,P38 MAPK,Pias,PP2A,Proteasome,RCAN1,RNA polymerase II,Sod,SOD2,Stat3-Stat3,Ubiquitin,VCAM1,Vegf	19	14	Cardiovascular System Development and Function, Cell Morphology, Cellular Development
8	Actin,ADCY,Calmodulin,Ck2,ELF3,ERK,FSH,G,G alphai,GABBR1,GBP2,Gpcr, HISTONE,IgG,Integrin,ISG20,Jnk,Lh,Mapk,NFKB1,Nos,PDPN,Pka,Pkc(s),PLA2,PLC, Pld,PLD1,Rap1,Ras homolog,Sapk,SAT1,SDC4,TCR,ZP3	14	10	Antigen Presentation, Cellular Assembly and Organization, Hematological System Development and Function
9	LRRN3,MIR29B1,MIR29B2,MIR29C (includes EG:407026),MIR363 (includes EG:574031),MIRLET7E (includes EG:406887)	2	1	Genetic Disorder, Skeletal and Muscular Disorders, Cell Signaling
