# Supplementary material for: Inflammatory Gene Regulatory Networks in Amnion Cells Following Cytokine Stimulation: Translational Systems Approach to Modeling Human Parturition
Source: PLoS One. 2011 Jun 2;6(6):e20560. doi: 10.1371/journal.pone.0020560 (PMC3107214; doi:10.1371/journal.pone.0020560)
Supplement: Table S8 — Focus genes and top functions from the network analysis, per network, for genes differentially expressed in TIL versus TNL patients. (PDF) [file pone.0020560.s011.pdf]

Table S8. Focus genes and top functions from the network analysis, per network, for genes differentially expressed in TIL versus TNL patients.

No.	Molecules in Network	Score	Focus Genes	Top Functions
1	ADCY,AREG,AXL,CAMK2N1,EMP1,Eotaxin,EREG,ERK1/2,FKBP5,FSH, G alphai,HAS1,hCG,HCK,HOXB7,IL8,IL23,Lh,Mek,MT1H,MT1X,MT2A,MYCBP2, NAMPT, PDGF BB,PHLDA1,PLAUR,PLC gamma,PLIN3,PTPRR,RCAN1,RGS16, SAA@,UPP1,Vegf	35	22	Cardiovascular System Development and Function, Cell Morphology, Cellular Development
2	CCL3,CCL4,CCL20,CCR1,CHEMOKINE,CSF3,CXCL1,CXCL2,CXCL3,CXCL5,CXCL6,Cyclooxygenase,Elastase,ELL2,Fibrinogen,FPR1,G0S2,GFPT2,Ikk (family),IL1, IL17R,IL1A,IL1RL1,IL1RN,LYMPHOTOXIN-ALPHA1-BETA2,Mmp,NFkB (complex), NfkB-RelA,NfkB1-RelA,Pro-inflammatory Cytokine,PTX3,SERPINA1,Tlr,TLR2, TNFAIP3	33	21	Cellular Movement, Hematological System Development and Function, Immune Cell Trafficking
3	ALP,BHLHE40,BMP2,Calmodulin,CASP6,CDKN1A,COL1A2,Collagen type I, CTBP2,Cyclin A,Cyclin E,E2f,Estrogen Receptor,Hdac,Histone h4,Hsp27,IER3, MORF4L2,MRAS,MXD1,N4BP2L2,NFIB,Notch,Nuclear factor 1,Pdgf,PI3K, PITPNA,Rb,RB1,RNF144B,Rxr,Smad,SMARCA2,VitaminD3-VDR-RXR,ZNF160	24	17	Cell Cycle, Cellular Development, Embryonic Development
4	ARHGAP1,ARHGAP12,BET1,BMX,BNIPL,CDC42,DEFA1 (includes EG:1667), DOCK9,EGF,FABP7,FAM84A,FBXO42,FCGR2C,FRYL,G,G-protein gamma, HMGN3,Il8r,IL8RB,LUC7L3,MIR199A1,MTAP (includes EG:4507),NFYB,Pld, RHOA,RhoGap,SDC1,SFRS1,SFRS5,SH3BP1,SRC,TRIO,Vegf Receptor, ZNF468,ZNF611	20	15	Cellular Movement, Embryonic Development, Hair and Skin Development and Function
5	Ap1,BCL2A1,Cbp/p300,CD3,DIO2,DSG3,GADD45B,HLA-DR,HMOX1,Hsp70, ICAM1,IFN Beta,Ifn gamma,IgG,IL6,IL12 (complex),Interferon alpha,LDL,MARCH6, Nfat (family),NFKB1,Nos,NUMBL,P38 MAPK,PLIN2,Sapk,SOCS3,SOD2,STAT, Stat3-Stat3,STAT5a/b,TAGLN,Tgf beta,TINF2,TNIP1	20	16	Cell Death, Liver Necrosis/Cell Death, Cellular Compromise
6	AGL,BMPR1A,C3ORF63,CEACAM1,CTNNB1,DLX5,EGFR,ERRFI1,FAT1,FLRT3, HGS,HMOX1,LIMCH1,MARK1,MIR124-1,NLK,POU5F1,SFN,SMAD7,SPIRE1, TNS4,TOM1L1,UBAP1,UBC,UNC84B,USP48,USP9X,VCAN,VIP,WWP1,WWTR1, XDH,ZNF259,ZNF302,ZNF638	20	15	Cellular Development, Cellular Growth and Proliferation, Tissue Morphology
7	26s Proteasome,ABLIM2,Akt,ASNS,C19ORF2,Caspase,Ck2,CLDN1, ERK, Focal adhesion kinase,F Actin, HELZ,Histone h3,Ikb,IKK (complex),Il8r,Jnk,KSR2,Mapk, NFKBIA,NKX3-1,NOD1,ODC1,Pka,Pkc(s),PRKDC,Rap1,Ras,RNA polymerase II,RWDD3,TCF7L2 (includes EG:6934),TJP1,Ubiquitin,UBR5,USP9X	19	14	Developmental Disorder, Organ Morphology, Reproductive System Development and Function

Only the top 7 scoring networks are depicted.
